# Comprehensive mapping of Arabidopsis alternative splicing landscape reveals key insights into plant development and immunity

**DOI:** 10.1002/tpg2.70022

**Published:** 2025-03-28

**Authors:** Teura Barff, Ingrid Berenice Sanchez Carrillo, Valeria Paola Parra Gutiérrez, Mélodie B. Plourde, David L. Joly, Hugo Germain

**Affiliations:** ^1^ Department of Chemistry, Biochemistry and Physics and Groupe de Recherche en Biologie Végétale Université du Québec à Trois‐Rivières Trois‐Rivières Québec Canada; ^2^ Département de biologie Université de Moncton Moncton New Brunswick Canada

## Abstract

The different steps of alternative splicing (AS) in plants and its regulatory mechanisms have already been studied extensively. Its broader impact on cell identity, plant immunity‐related genes, and their study as a whole remains to be investigated. Using transgenic plants, we sorted 11 different *Arabidopsis thaliana* cell types ranging from root to aerial organs using fluorescence‐activated cell sorting. RNA‐seq data were analyzed with vast‐tools and enabled us to generate a high‐resolution AS landscape across multiple cell types, all collected through the same experimental procedure. The analysis of cell type‐specific gene expression and alternative splicing events highlights the importance of AS on transcription and AS regulation itself. AS is also shown to be tightly linked to cell identity. By using closely related cell types, we captured alternative splicing events involved in specific stages of plant development. The columella cells, among others, show intensified AS regulation and an interesting splicing profile, especially regarding immunity‐related genes. Overall, our analysis brings a valuable tool in the study of cell type identity, plant immunity, and AS.

## Core Ideas

- Alternative splicing plays a role in regulating transcription.
- Both alternative splicing and gene deregulation influence distinct sets of genes.
- Alternative splicing is closely associated with determining cell identity.

**Abbreviations**
AS: alternative splicing
DEG: differentially expressed cell type‐specific genes
DEX: dexamethasone
DSE: differentially spliced cell type‐specific event
DSG: differentially spliced cell type‐specific genes
PSI: percentage spliced‐in value

## 1 INTRODUCTION

Alternative splicing (AS) is the process by which a single gene produces multiple messenger RNAs. It is believed that AS provides eukaryotes the level of transcriptome and proteome complexity necessary to adapt to environmental cues (Chaudhary et al., 2019; Reddy et al., 2013). This widespread process is well documented in mammals and higher plants. In the human genome, it is estimated that approximately 94% of multiexonic genes undergo AS (Wang et al., 2008), while plants are estimated to alternatively splice up to 62% of multiexonic genes under normal developmental conditions (Marquez et al., 2012; Martín et al., 2021). The four main types of alternative splicing events (ASEs) are exon skipping (EX), intron retention (IR), alternative donor site (ALTD), and alternative acceptor site (ALTA). Intron retention is the most prevalent type of ASE in plants, with up to 65% of intron‐containing genes subjected to AS (Marquez et al., 2012; Ner‐Gaon et al., 2004), as opposed to 5% in humans (Wang et al., 2008). AS may lead to (i) translation into functional proteins, thus diversifying proteome complexity, (ii) introduction of a premature terminal codon leading to degradation through nonsense‐mediated mRNA decay (NMD) (Drechsel et al., 2013; Kalyna et al., 2012; Ohtani & Wachter, 2019), (iii) nuclear sequestration for further mRNA processing on demand (Göhring et al., 2014; Hartmann et al., 2018; Yang et al., 2017), and (iv) translation into truncated proteins (Liu et al., 2013). While AS regulation mostly relies on splicing factors like serine–arginine‐rich (SR) proteins (Erkelenz et al., 2013; Hertel & Maniatis, 1999) and heterogeneous nuclear ribonucleoproteins (hnRNPs) (Huelga et al., 2012), AS is considered a co‐transcriptional process, thus it will be greatly impacted by chromatin structure and by the speed and phosphorylation state of the mRNA polymerase II (Zhu et al., 2018).

Evidence of the impact of AS in plant development and cell type differentiation keeps emerging. While uncovering the role of specific AS isoforms in a precise stage of plant growth has been the focus of many, the advancement of high‐throughput transcriptomic analyses has enabled the mapping of the plant AS landscape to become increasingly common. For example, the AS mapping of the potato (*Solanum tuberosum* L.), the black gram (*Vigna mungo*), and even the cotton (*Gossypium hirsutum*) were recently published (Hazra et al., 2023; Ogungbayi et al., 2023; Zhang et al., 2022). *Arabidopsis thaliana* (Arabidopsis) is one of the most studied plant species, including when it comes to AS research. High‐resolution databases compiling the AS landscapes of Arabidopsis have brought valuable information and tools to understand the global impact of plant AS (Guo et al., 2023; Liu et al., 2023; Martín et al., 2021; Zhang et al., 2022). Some studies focused on AS across cell types (Li et al., 2016; Martín et al., 2021; Misra et al., 2023), while others focused on AS under various conditions like abiotic and biotic stresses (Guo et al., 2023; Martín et al., 2021), the latter highlighting the broader importance of AS when facing immune challenges.

Among all the factors that influence plant immunity, the impact of cell identity has until recently been seldom investigated. The interest in organ‐specific defense response emerged with the need to understand colonization patterns of naturally occurring pathogens. Some pathogens will favor given organs while others are able to infect the entire host (Balmer & Mauch‐Mani, 2013). One of the first studies recording organ‐specific disease was done on the oomycete *Hyaloperonospora arabidopsidis*. During incompatible interaction, *H. arabidopsidis* causes resistant‐like symptoms in Arabidopsis leaves but not in roots, although the plant still expresses the immune regulators *EDS1* and *NPR1* in both plant parts (Hermanns et al., 2003). Although evidence of organ‐specific immune response has been mounting in the past two decades, the study of cell type‐specific immunity‐related genes is still at its starting point.

For example, the perception of the flagellin peptide flg22 by multiple Arabidopsis root cell types was shown to be sufficient to trigger PAMP‐Triggered Immunity (PTI); however, the intensity of the triggered response varies according to the cell type (Wyrsch et al., 2015). In more detail, flg22 and the plant elicitor peptide 1 (Pep1) induce a different transcriptional network in the root epidermis, cortex, and pericycle by modulating the expression of genes with various transcription factor binding motifs (Rich‐Griffin et al., 2020). Very recently, Tang et al. (2023) characterized the Arabidopsis leaf cell type‐specific responses to *Colletotrichum higginsianum* infection using single‐cell transcriptomics, further asserting the importance of cell identity on plant immunity.

Many steps of the plant defense response are paired with AS of resistance‐related transcripts. For instance, PTI induction will trigger AS of the CPK28 transcript, leading to the accumulation of a nonfunctional truncated CPK28 protein, thus alleviating its role as a negative regulator of plant defense (Dressano et al., 2020). Similarly, the resistance gene *RPS4* from Arabidopsis, which confers resistance to *Pseudomonas syringae* pv *tomato* strain DC3000 (DC3000) expressing AvrRps4, is also subjected to AS. More specifically, removal of one or two of the introns found in the dominant splice variant of *RPS4* did not affect its expression but suppressed the function of the protein it encodes. This suggests that multiple variants of the RPS4 protein are required for proper immune response (Zhang & Gassmann, 2003). Phytopathogens are also able to modulate the host splicing machinery, as is the case for the *Phytophthora sojae* effector PsAvr3c (Huang et al., 2017). Those are just a few examples showing the importance of proper mRNA splicing in plant defense.

Despite evidence of cell identity, immunity‐related genes, and AS being interconnected, very little effort has been invested so far to study these processes as a whole. While extensive mapping of the Arabidopsis AS landscape has already been performed, these databases were generated using publicly available aggregated datasets from varying sources. The isolation of different cell types is a limiting step, as the number of cell types varies from one developmental stage to another, rendering this process arduous. Furthermore, techniques like manual organ dissection and laser‐capture microdissection can be less precise and unpractical. In this study, we used cell type‐specific reporter transgenic lines coupled with fluorescence‐activated cell sorting (FACS) to collect 11 different Arabidopsis cell types from roots, stem, and leaves. In an effort to uncover the impact of AS on cell identity and immunity‐related genes, we compared gene expression and alternatively spliced events across these 11 different cell types with a particular focus on immunity‐related transcripts.

Our results demonstrate the feasibility of a pipeline combining flow cytometry sorting of fluorescent protoplasts from 11 cell types, followed by RNA extraction and deep sequencing. We observe that gene expression is poorly connected with AS and successfully identify deregulated genes and AS events that are specific to individual cell types or groups of cell types. We also uncover the previously unreported importance of AS in regulating immunity‐related genes, more specifically in columella cells. The use of closely related cell types enabled us to capture subtle changes in AS regulation needed during development and immune regulation. Overall, the dataset generated here provides a valuable tool in the study of cell type identity, plant immunity‐related genes, and AS.

## 2 METHODS

### 2.1 Plant material and growth conditions

The *Arabidopsis thaliana* Col‐0 transgenic plants used in this study were kindly provided by Pr. Sebastian Wolf from Universität Heidelberg (Schürholz et al., 2018) and are listed in Table 1. Plants were grown on Murashige and Skoog (MS) medium (2.15 g/L MS basal salt mixture [Phytotech], 1% [w/v] agar, and pH 5.8) supplemented with 30 µM dexamethasone (DEX) (Sigma‐Aldrich). Once sowed, plates were placed in growth chambers with 10‐/14‐h light/dark cycles. To ensure enough cells from each cell type were present, transgenic lines harboring aerial cell type‐specific promoters were grown for longer than the ones with root cell type‐specific promoters. Therefore, p*LTP1*, p*UFO*, p*REV*, p*ML1*, and p*APL* were incubated horizontally for 21 days, while p*ATHB8*, p*SCR*, p*TMO5*, p*SMLX5*, p*PXY*, and p*CASP1* transgenic lines were incubated vertically for 14 days.

**Core Ideas**

- Alternative splicing plays a role in regulating transcription.
- Both alternative splicing and gene deregulation influence distinct sets of genes.
- Alternative splicing is closely associated with determining cell identity.

**TABLE 1:** Cell type‐specific promoters used in this study.

Category	Promoter	Gene	Gene ID	Cell type	Reference
Aerial	p*ML1*	MERISTEM LAYER 1	AT4G21750	L1 Layer epidermis	(Session et al., 1999)
	p*REV*	REVOLUTA	AT5G60690	Shoot apical meristem (SAM) central zone	(Otsuga et al., 2008)
	p*UFO*	UNUSUAL FLORAL ORGANS	AT1G30950	SAM peripheral zone	(Hepworth et al., 2006)
	p*LTP1*	LOCALIZED LIPID TRANSFERT PROTEIN1	AT2G38540	Epidermis in stem	(Thoma et al., 1994)
Root	p*APL*	ALTERED PHLOEM DEVELOPMENT	AT1G79430	Phloem (differentiating)	(Bonke et al., 2003)
	p*SCR*	SCARECROW	AT3G54220	Endodermis, quiescent center (QC) in the root apical meristem (RAM), starch sheath in stem	(Malamy & Benfey, 1997)
	p*TMO5*	TARGET OF MONOPTEROS5	AT3G25710	Xylem precursor	(De Rybel et al., 2013)
	p*PXY*	PHLOEM INTERCALATED WITH XYLEM	AT5G61480	(Pro)cambium	(Wang et al., 2013)
	p*CASP1*	CASPARIAN STRIP MEMBRANE DOMAIN PROTEINS	AT2G36100	Differentiating endodermis	(Roppolo et al., 2011)
	p*SMXL5*	SUPPRESSOR OF MAX2 1‐LIKE5	AT5G57130	Phloem (precursors)	(Wallner et al., 2017)
	p*ATHB‐8*	HOMEOBOX GENE 8	AT4G32880	Procambium, xylem precursors and columella in RAM	(Baima et al., 1995)

### 2.2 Protoplast extraction

Protoplasts were extracted from the above‐mentioned seedlings following the protocol described by Bargmann and Birnbaum (2010) and Sanchez Carrillo et al. (2023) with the following modifications. For each Arabidopsis transgenic line, sections of plants containing the necessary cell type were cut from 30 MS plates with a minimum of 200 seedlings per plate. The sections were incubated in protoplasting solution (1% cellulase from *Trichoderma* sp. [Sigma‐Aldrich], 0.3% Macerozyme [Sigma‐Aldrich], 0.4 M mannitol, 20 mM KCl, 20 mM MES, 10 mM CaCl_2_, 5 mM β‐mercaptoethanol, and 0.1% BSA) for 90 min on an orbital shaker at 75 rpm. Protoplasts were passed through a 30 µm filter, centrifuged for 10 min at 500 *g*, and resuspended in fresh W5 buffer (154 mM NaCl, 125 mM CaCl_2_, 5 mM KCl, 2 mM MES, and pH 5.7). To ensure mTurquoise2 expression remained after extraction, protoplasts were checked under confocal microscopy before proceeding to FACS.

### 2.3 Confocal microscopy

mTurquoise2 expression was observed on seedlings prior to protoplast isolation with a Leica TCS SP8 confocal laser scanning microscope (Leica Microsystem). Seedlings were stained with 1 ng/mL propidium iodide (PI) for 30 min before mounting in water under a coverslip. Excitation/Emission wavelengths during acquisition were 405 nm/460–520 nm for mTurquoise2 and 488 nm/590–660 nm for PI (Figure S1). Protoplasts before and after cell sorting were also visualized for mTurquoise2 expression using the same excitation and emission wavelength mentioned above (Figure S2A,B).

### 2.4 Fluorescence‐activated cell sorting

FACS was performed on freshly extracted protoplasts using the BD FACS Melody Cell Sorter with a 100 µm nozzle and W5 buffer as sheath fluid. Flow rate was 1%, and event rate was kept under 6000 events/s. The BD FACS Melody Cell works with an operating pressure of 23 psi and a drop frequency of 34 kHz. mTurquoise2‐positive protoplasts were identified by plotting events obtained with a laser of 405 nm and a bandpass filter of 528/545 nm against events obtained with a laser of 488 nm and a bandpass of 527/532. For each experiment, protoplasts extracted from transgenic plants grown on MS media without DEX were used to differentiate mTurquoise2 fluorescence from autofluorescence (Figure S2C–H). A total of 50,000–100,000 cells were sorted and collected in three to six replicates for each cell type. Samples were harvested at 4°C directly in RLT lysis buffer (Qiagen) and stored at −80°C. In total, 48 samples were collected and processed for sequencing (Table S1).

### 2.5 RNA isolation and sequencing

RNA was extracted with the RNeasy Micro kit (Qiagen), with the recommended DNase treatment according to the manufacturer's protocol. Quantification was performed with the Quanti‐iT Ribogreen RNA reagent kit (Invitrogen) using the manufacturer's low‐range standard curve. The Synergy H1 Multimode microplate reader (Biotek) was used to observe the Quant‐iT Ribogreen RNA reagent's fluorescence when bound to RNA, with an excitation/emission wavelength of 485/520 nm. QC was done with a 2100 Bioanalyzer (Agilent). Libraries were generated with a low input RNA Library Prep kit for Illumina (NEB) and sequenced on an Illumina NovaSeq 6000 S4 system in paired‐end reads (100 bp) by the *Centre d'expertise et de services Génome Québec*.

### 2.6 RNA‐seq data analysis

Over 2 billion raw reads were trimmed using Trimmomatic v0.39 (Bolger et al., 2014) to remove the sequencing adapters. The alignment was performed with vast‐tools v2.2.0 (Irimia et al., 2014; Tapial et al., 2017). This step is executed within vast‐tools with bowtie 1.0.0 (Langmead et al., 2009). Trimmed reads were mapped to the Arabidopsis (araTha10 version 23.06.20) library from PASTDB. This database is composed of exon–exon and exon–intron junctions annotations generated using TAIR10, Ensembl Plants v31, and a collection of data as scaffold (Martín et al., 2021). The vast‐tools alignment tool slices reads to 50 nt fragments with a sliding window of 25 nt to increase the mapping of junctions. This step consists of several modules that enable the identification of exon skipping, intron retention, and alternative donor and acceptor events (Tapial et al., 2017).

### 2.7 Alternative splicing analysis

All of the AS analyses were done using the vast‐tools v2.2.0 toolset. By mapping reads to exon–exon junctions and exon–intron junctions, vast‐tools is able to generate the percentage spliced‐in value (PSI) (Figure S3). Only the splicing events with sufficient read coverage (above or equal to “LOW” coverage described by Martín et al. 2021) in at least 10 samples across cell types were considered.

Furthermore, only the samples that passed the binomial test for IR were kept for further analysis. This test assesses proper balance of read at the exon–intron junction and is used to filter out IR events with read imbalance as described in Braunschweig et al. (2014). The script used to identify cell type‐specific ASE was developed by Martín et al. (2021) and is available at https://github.com/vastdb‐pastdb/pastdb. At this step, root and aerial cell types were analyzed separately (Table 1). For the different cell types to be included in the analysis, each group needed a minimum of two replicates with sufficient coverage, and the minimum number of cell types with sufficient coverage from each organ was four. To be considered cell type‐specific, (i) a splicing event required an average |∆PSI| of at least 25 when compared to the average of all other samples from either root or aerial cell types (|global ∆PSI| > 25). Moreover, (ii) the ASE needed a difference in average PSI of at least 15 when compared to any other cell type (|minimum ∆PSI| > 15). Finally, the minimum PSI range (i.e., the difference between the maximum value from a cell type with the maximum value from another) for the event to be considered AS among groups was 2.

The immunity‐related gene list was compiled using the “defense response” annotation and children terms as a filter through the gene ontology (GO) annotation of the *Arabidopsis* list from TAIR (predicted and experimental) (Berardini et al., 2004). This screening led to 4869 annotations and 2822 genes (Table S2).

### 2.8 Differential gene expression assessment

The differential gene expression step was executed with a vast‐tools script built by Martín et al. (2021) (Figure S3). The script is accessible at the following link: https://github.com/vastdb‐pastdb/pastdb. For this analysis, aerial and root cell types were separated and only genes with a median expression cRPKM ≥ 5 in at least one cell type were considered. A gene with an absolute fold change ≥2 when compared to the median of each cell type, a fold change ≥5 when compared to the global median of all the other conditions, and an absolute difference in cRPKM ≥ 2 with every other cell type would be considered cell type specific. The normalized counts of the genes regulated by the cell type‐specific promoters (Table 1) were calculated with DESeq2 (Love et al., 2014) (Figure S4C).

### 2.9 GO term analysis

The functional annotation analysis was done with the DAVID tools v2023q2 (Huang et al., 2009; Sherman et al., 2022) using a custom background set of genes. This set is constituted of genes that pass the same quality control criteria used to define the cell type‐specific AS or genes.

## 3 RESULTS

### 3.1 Cell type identity is conserved upon cell sorting

**FIGURE 1:**
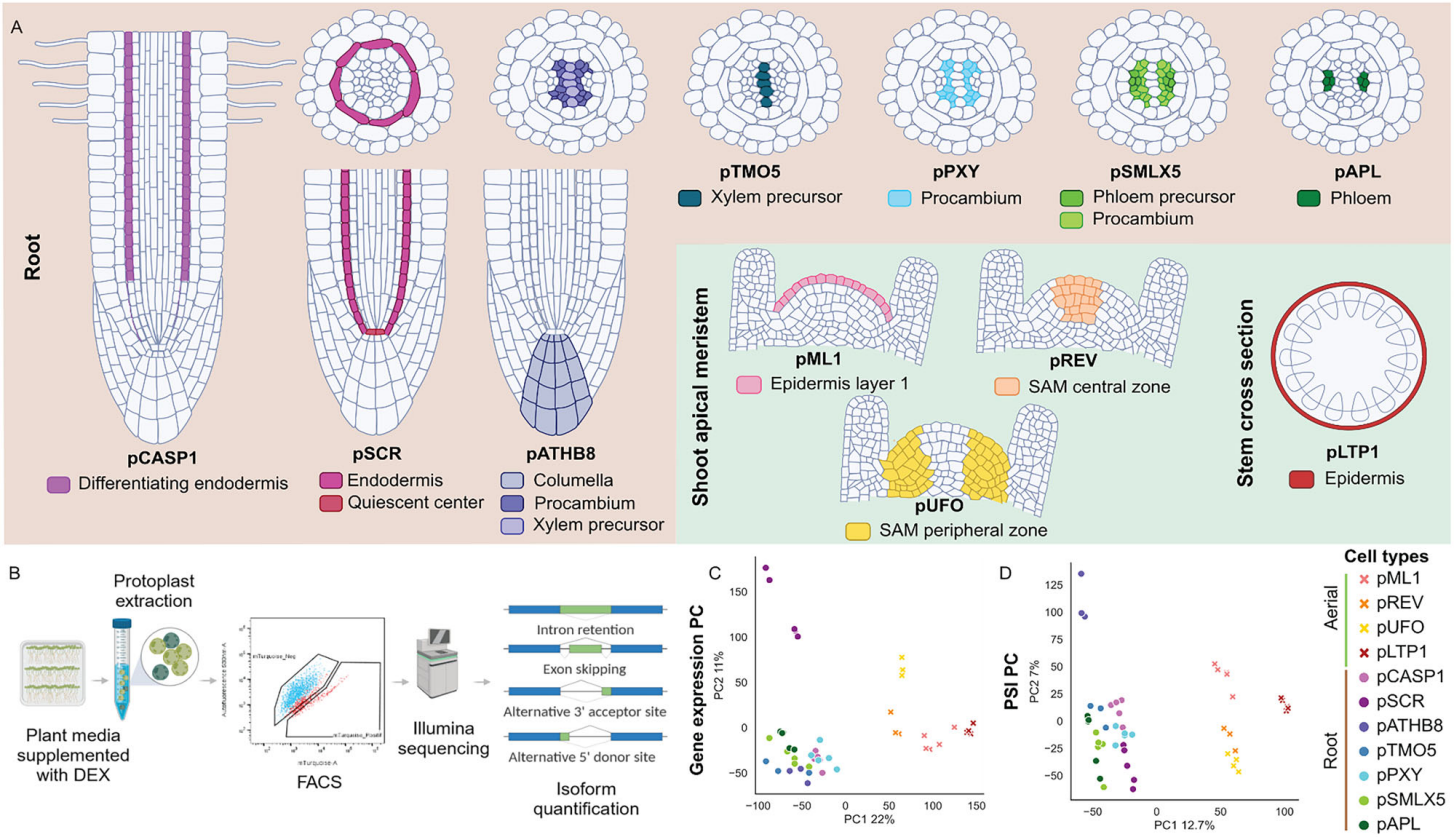
Cell type identity is conserved upon cell sorting. (A) Schematic representation of the cell types selected for this study. Green zone represents cell types found in aerial plant parts. Brown zone represents cell types in roots. Root cells are depicted either in the full root or in cross section. (B) Experimental workflow used to collect 11 different cell types of *Arabidopsis thaliana* for AS analysis. Plants are grown on a dexamethasone (DEX)‐containing medium and used for protoplast extraction. Protoplasts are then sorted through fluorescence‐activated cell sorting (FACS). To select only targeted cell types expressing mTurquoise2, protoplasts from plants grown on medium without DEX and therefore not producing mTurquoise2, are used as controls. The gating strategy to sort mTurquoise2‐positive cells shown here is of p*SMLX5*. Following FACS, sorted protoplast are proceeded to RNA extraction, library preparation and sequencing. The principal component analysis (PCA) done on (C) cRPKM of genes and (D) percentage spliced‐in values (PSIs) of splicing events that passed the quality filters described in Section 2.

The transgenic plants of Arabidopsis Col‐0 used here carried the gene encoding for the fluorescent protein mTurquoise2 (Goedhart et al., 2012), under the control of the inducible promoter p*OP*. This promoter relies upon the binding of the chimeric transcription factor LhG4. The interaction between LhG4 and the glucocorticoid DEX will cause the relocation of LhG4 to the nucleus, where it is able to bind pOP and activate it (Craft et al., 2005). The cell type‐specificity aspect of this system comes from the expression of LhG4, which is controlled by a cell type‐specific promoter (Figure 1A) (Schürholz et al., 2018). This multilayered system has enabled us to work with 11 different Arabidopsis transgenic lines, each harboring a different cell type‐specific promoter. To collect only the targeted cell types, the transgenic plants were grown on DEX‐containing medium (Figure 1B) and then used for protoplast extraction followed by sorting via FACS (Figure 1B). To prevent protoplast dedifferentiation, we acted in a timely fashion while still making sure that enough material was collected to proceed to FACS. The cell types subjected to Illumina sequencing are spread across the roots and aerial parts of the plants (Figure 1A, Table 1).

To ensure that the experimental design of this study is reliable, we first confirmed that extracting protoplasts and sorting them would have minimal effect on the integrity and identity of the cells. The confocal images of protoplasts taken before and after cell sorting confirmed that protoplast extraction and cell sorting did not affect mTurquoise2 emission (Figure S2A). They also confirmed that the protoplast population collected through FACS was indeed enriched with mTurquoise2‐emitting cells (Figure S2A,B). Both the confocal images and the FACS data assured that protoplasts do conserve their integrity and therefore can be used for RNA sequencing. With the RNA‐seq data gathered, we were able to calculate the PSI of each ASE and the transcripts cRPKM (corrected for mappability) using vast‐tools. After applying the quality filters described in Section 2, we performed a principal component analysis (PCA) with the transcripts’ cRPKM values (Figure 1C) and the ASEs PSI (Figure 1D). This analysis shows that the different cell types cluster together according to the organ from which they originate. The clustering of the replicates is also visible on the Spearman cluster heatmap (Figure S4A,B). In addition to the heatmap and PCA, we also looked at the normalized read counts of the genes natively controlled by the cell type‐specific promoters used in this study (Table 1, Figure S4C). Most of the genes observed exhibit the highest counts in their respective condition. Although *REV*, *LTP1*, *PXY*, *CASP1*, and *APL* did not demonstrate the highest level of counts in the cell type they belong to, they do harbor the second to fourth highest count numbers. This can be explained by the fact that some of those genes are expressed in similar cell types. *LPT1*, for instance, has the highest count number in the cell type represented by p*ML1*, which is expected as both *ML1* and *LTP1* are expressed in the epidermis (Han et al., 2020; Sessions et al., 1999; Thoma et al., 1994). It is also the case for *CASP1*, which has the highest count number in p*SCR*. While being expressed in the quiescent center, *SCR* is also expressed in the endodermis like *CASP1* (Malamy & Benfey, 1997; Roppolo et al., 2011). *SMLX5* and *APL* are both specific to phloem cells, which is reflected by *SMLX5* having the highest number of counts in p*APL* (Bonke et al., 2003; Wallner et al., 2017). These results demonstrate that the procedure used herein does not significantly alter cell identity and can then be used to enrich cell populations for further characterization.

### 3.2 Gene expression is uncoupled from alternative splicing

**FIGURE 2:**
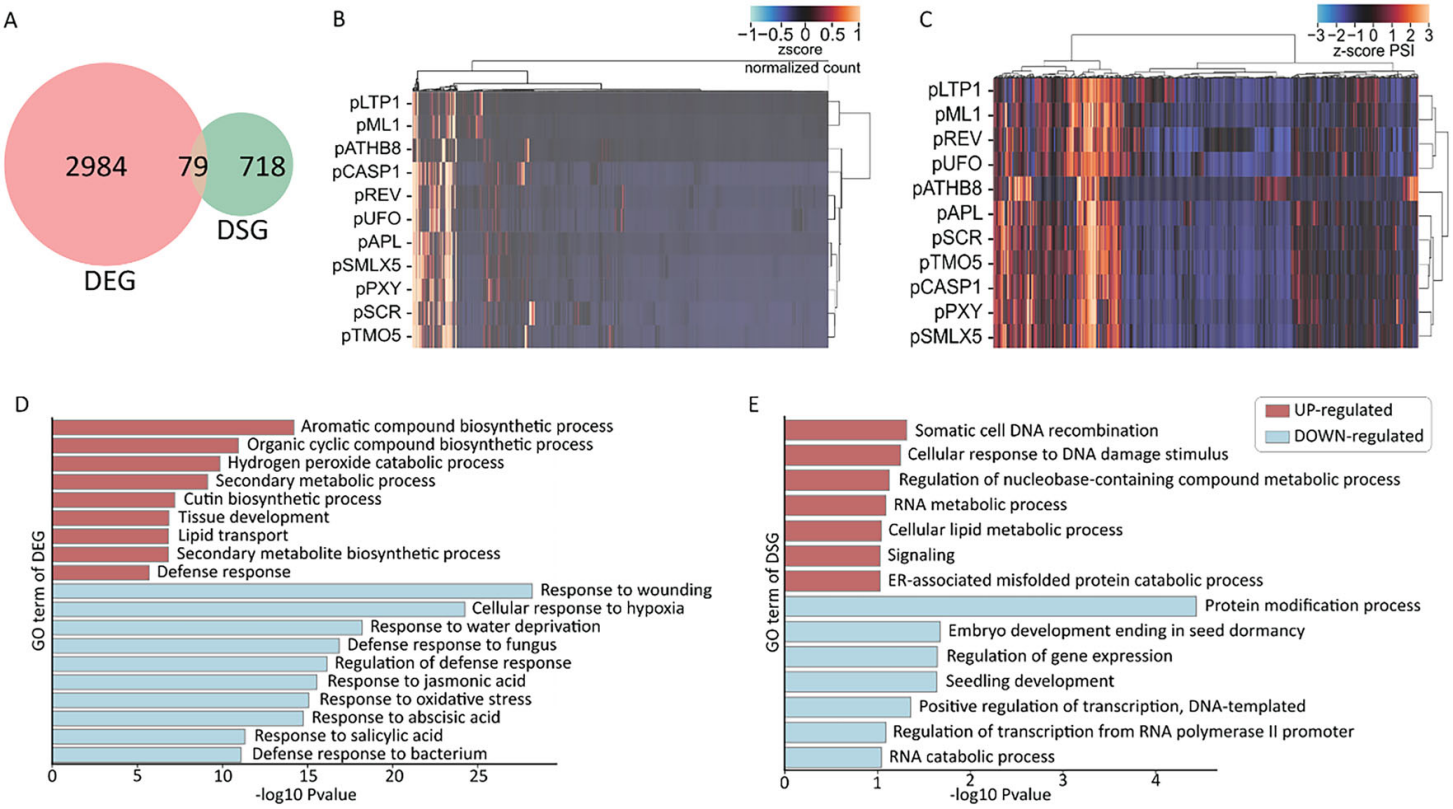
Gene expression is uncoupled from alternative splicing. (A) Venn diagram of differentially expressed cell type‐specific genes (DEGs) and differentially spliced cell type‐specific genes (DSGs). Genes with at least one alternatively spliced event (ASE) were considered a DSG. *z*‐Score cluster heatmap of (B) normalized counts from DEG and (C) PSIs from differentially spliced events (DSEs). Clustering was done using the ward method. The conversion to *z*‐score was performed after the hierarchical clustering so that it only affects the color grading. Enriched gene ontology (GO) terms in DEGs (D) and DSGs (E). Red and blue indicates up‐ and downregulated genes. Bars indicate −log10 of the DAVID's EASE score modified *p*‐value.

To assess differences in gene expression in a cell type‐specific manner from aerial or root organs, the script performed pairwise comparisons between the target cell type and a pool of all the other cell types. The goal here was to uncover genes whose expression did not overlap between cell types. Similarly, differences in gene splicing between cell types were measured by comparing PSIs in a targeted tissue against the average of all the other tissues, as described by Martín et al. (2021). These two analyses yielded 2984 and 718 differentially expressed cell type‐specific genes (DEGs) and differentially spliced cell type‐specific genes (DSGs) (Table S3) (Figure 2A). The two sets only share 79 genes, which are both DEG and DSG. Overall, the sets of genes that are regulated at the transcriptional level and the AS level show only minimal overlap, suggesting the two processes act independently in managing cell identity. For a gene to be considered differentially spliced in a cell type‐specific manner, it needed at least one differentially spliced cell type‐specific event (DSE). From the 718 DSGs, 997 DSEs were identified, implying that some genes have more than one cell type‐specific DSE. The DEGs (Figure 2B) and DSEs (Figure 2C) exhibit distinct profiles. Interestingly, the splicing profiles also cluster according to the organ of origin, contrasting with the expression profiles, for which no evident pattern can be discerned (Figure 2B,C). Even though the two groups are strikingly different, a unique pattern can be observed for p*ATHB8* (Figure 2B,C). We also looked at the expression of various housekeeping genes in p*ATHB8* and ensured their expression remains unchanged compared to all the other cell types (Table S4) (Czechowski et al., 2005).

The functional annotation analysis of each set of the DEG and DSG shows very little similarity (Figure 2D,E). Among the processes upregulated at the gene expression level, we notice tissue development. On the other hand, the downregulated processes are generally more focused on defense responses and stress (Figure 2D). The DSGs are enriched for processes like protein modifications and RNA/DNA metabolic process (Figure 2E). This is compatible with the fact that many splicing factors auto‐regulate themselves through AS (Lazar & Goodman, 2000; Lopato et al., 1999, 1996). Interestingly, annotations related to transcription regulation are also represented in the DSGs (Figure 2E), meaning that AS is involved in gene expression regulation. These results indicate that the regulation of transcription and of AS in a cell type‐specific manner are independent.

### 3.3 Cell identity shapes the need for transcription or alternative splicing regulation

**FIGURE 3:**
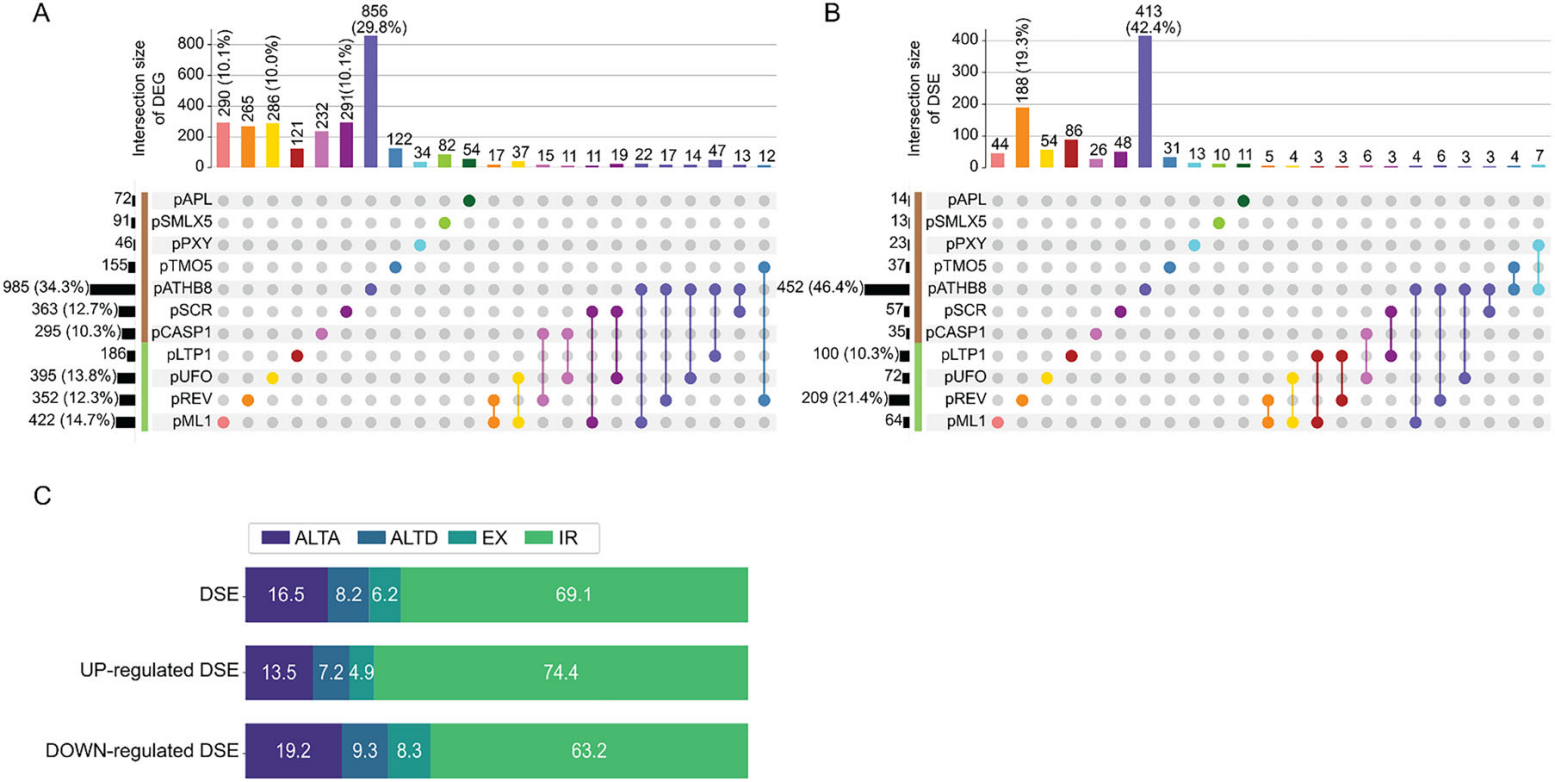
Cell identity shapes the need for transcription or alternative splicing regulation. Upsetplots of (A) differentially expressed cell type‐specific genes (DEGs) and (B) differentially spliced cell type‐specific events (DSEs). Each color represents a cell type. Vertical bars show the number of overlapping genes or ASEs across cell types. Colored dots indicate intersections from which DEGs and DSEs overlap. The number of genes or ASEs in each intersection is indicated over the vertical bars, percentage is displayed when above 10%. Horizontal bars represent the total number of genes or ASEs that are differentially expressed or spliced according to different cell types. Green and brown bars represent aerial and root cell types, respectively. Proportions of the types of ASE from the DSEs, the upregulated and downregulated DSE (C). Numbers in bars indicate the percentage of ASEs that are either alternative acceptor site (ALTA) (dark violet), alternative donor site (ALTD) (teal), exon skipping (EX) (dark green), or intron retention (IR) (light green).

The importance of AS in cell differentiation has already been studied (Li et al., 2016). To examine how the identity of this specific set of cell types affects the ASE population, we looked at the distribution of DEGs and DSEs across cell types. p*ATHB8*, a promoter mostly expressed in the procambium, xylem precursor, and columella in the root apical meristem, displays a notable distribution profile with the largest number of DEGs (985) (Figure 3A) and DSEs (452) (Figure 3B), regardless of whether they are up or downregulated. The cell types represented by p*ATHB8* make up 34% and 46% of the total DEGs and DSEs, respectively. Of the 985 DEGs in p*ATHB8*, 856 are unique to that promoter. Of the 129 genes left, 100 are shared between p*ML1*, p*REV*, p*UFO*, and p*LTP1*, which are from aerial organs. The other 29 genes are spread across the rest of the cell types (Figure 3A). At the other end of the spectrum, cells sorted with p*PXY* exhibited the smallest number of DEGs with only 46 (1.6%) deregulated genes (Figure 3A). At the splicing level, p*ATHB8* presents 452 DSEs, among which 413 are unique to that promoter, whereas p*SMLX5* has only 13 (1.3%), making those two the most and least prone to AS regulation, respectively (Figure 3B). Our analysis aimed at identifying nonoverlapping DSEs. Hence, p*APL* and p*SMLX5* both being expressed in the phloem led to the identification of fewer nonoverlapping DSEs between promoters. Interestingly, some cell types display twice as many DEGs as DSEs. This is the case for p*SMLX5*, p*CASP1*, p*SCR*, and p*ML1* (Figure 3A,B). This phenomenon is not observed for DSEs over DEGs, which can in part be explained by the fact that the overall number of observed DEGs is greater than the number of DSGs (2984 vs. 714). Nonetheless, p*LTP1* and p*REV* both have over 1.5‐fold more DEGs than DSEs (Figure 3A,B). This further implies that the importance of AS differs depending on the cell identity and the function they hold. The cell types represented by p*ATHB8*, for example, appear to host processes that require more intense transcriptional and posttranscriptional regulation. To have a better understanding of the impact of cell type‐specific AS, we looked at the proportion of the different types of events. As has been globally reported, the most prevalent type of ASE is intron retention, representing 69.1% of all the DSEs identified (Figure 3C). Intron retention is followed by alternative acceptor site, alternative donor site, and exon skipping (16.5%, 8.2%, and 6.2%, respectively). This corroborates previous reports of event type proportions found in plant cell type‐specific AS datasets (Li et al., 2016; Martín et al., 2021).

Even though the procedure to identify cell type‐specific genes and ASEs uncovers only nonoverlapping DEGs and DSEs, some are still shared between cell types. The analysis was done by separating cell types originating from different organs, which explains the overlap between cell types from aerial parts and from roots. The distribution of DEGs and DSEs across cell types highlights their varying needs in terms of splicing and transcription regulation.

### 3.4 Direction of gene expression and AS regulation is dependent on cell identity and function

**FIGURE 4:**
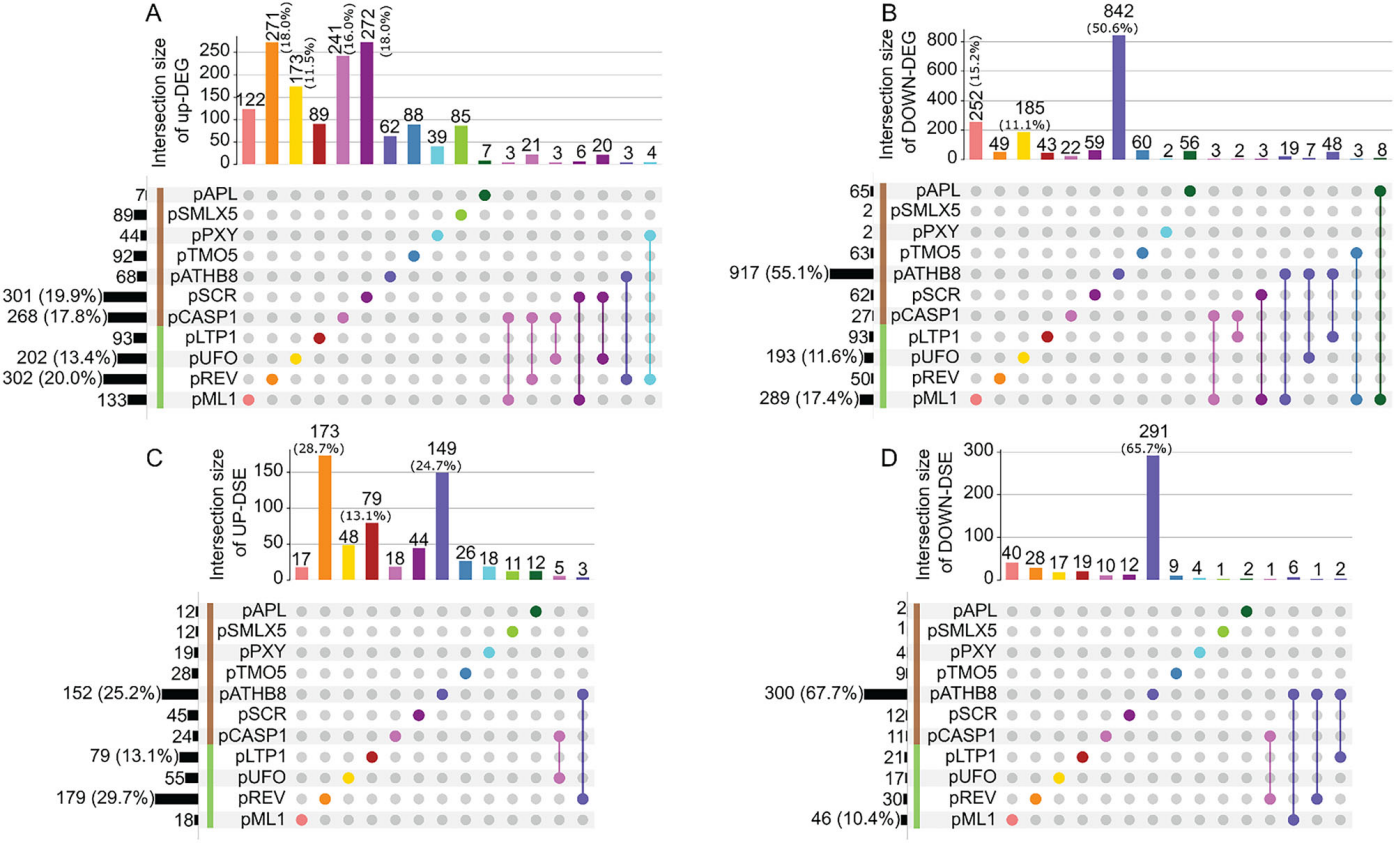
Direction of gene expression and AS regulation is dependent on cell identity and function. Distribution of (A) upregulated (log 2 fold change [FC] ≥ 2) and (B) downregulated (log 2 FC ≤ −2) differentially expressed DEGs, and of (C) upregulated (∆PSI > 25) and (D) downregulated DSEs (∆PSI < −25). As previously mentioned, cell types are colored as indicated; vertical bars represent the amount of genes or ASEs overlapping in the corresponding intersection. Numbers over the vertical bars indicate the quantity of genes or ASEs, percentage is displayed when above 10%. Green and brown bars represent aerial and root cell types, respectively.

When DEGs are separated according to their upregulation (Figure 4A) or downregulation (Figure 4B), we can observe that the majority of p*ATHB8* DEGs are downregulated with 62 up‐DEGs (∼4% of the total up‐DEGs) (Figure 4A) and 842 down‐DEGs (50.6% of the total down‐DEGs) (Figure 4B). Although not at the same scale, p*APL* also shows more down‐DEGs (56) (Figure 4B) than up‐DEGs (7) (Figure 4A). While the overall trend of the DEGs tends toward downregulation, this is mostly due to p*ATHB8*, as other cell types like p*SCR*, p*TMO5*, p*PXY*, p*LTP1*, p*SMLX5*, p*REV*, and p*CASP1* have mostly up‐DEGs. Among those mainly upregulated DEGs cell types, p*REV*, p*CASP1*, and p*SCR* are the most striking as they have 271 (18%), 241 (16%), and 272 (18%) of up‐DEGs (Figure 4A) and 49 (∼3%), 22 (∼1.2%), and 59 (∼3.4%) down‐DEGs (Figure 4B), respectively. Looking at the upregulated (Figure 4C) and downregulated (Figure 4D) DSEs, a clear tendency toward upregulation can be seen. The only exceptions are p*ATHB8* and p*ML1*, which respectively have 149 (24.7%) and 17 (2.8%) up‐DSEs (Figure 4C) and 291 (65.7%) and 40 (9%) down‐DSEs (Figure 4D). Those results indicate that the need for up‐ or downregulation of DEGs varies according to the identity and the function of each cell type.

### 3.5 Differential splicing of immunity‐related genes is cell type specific

**FIGURE 5:**
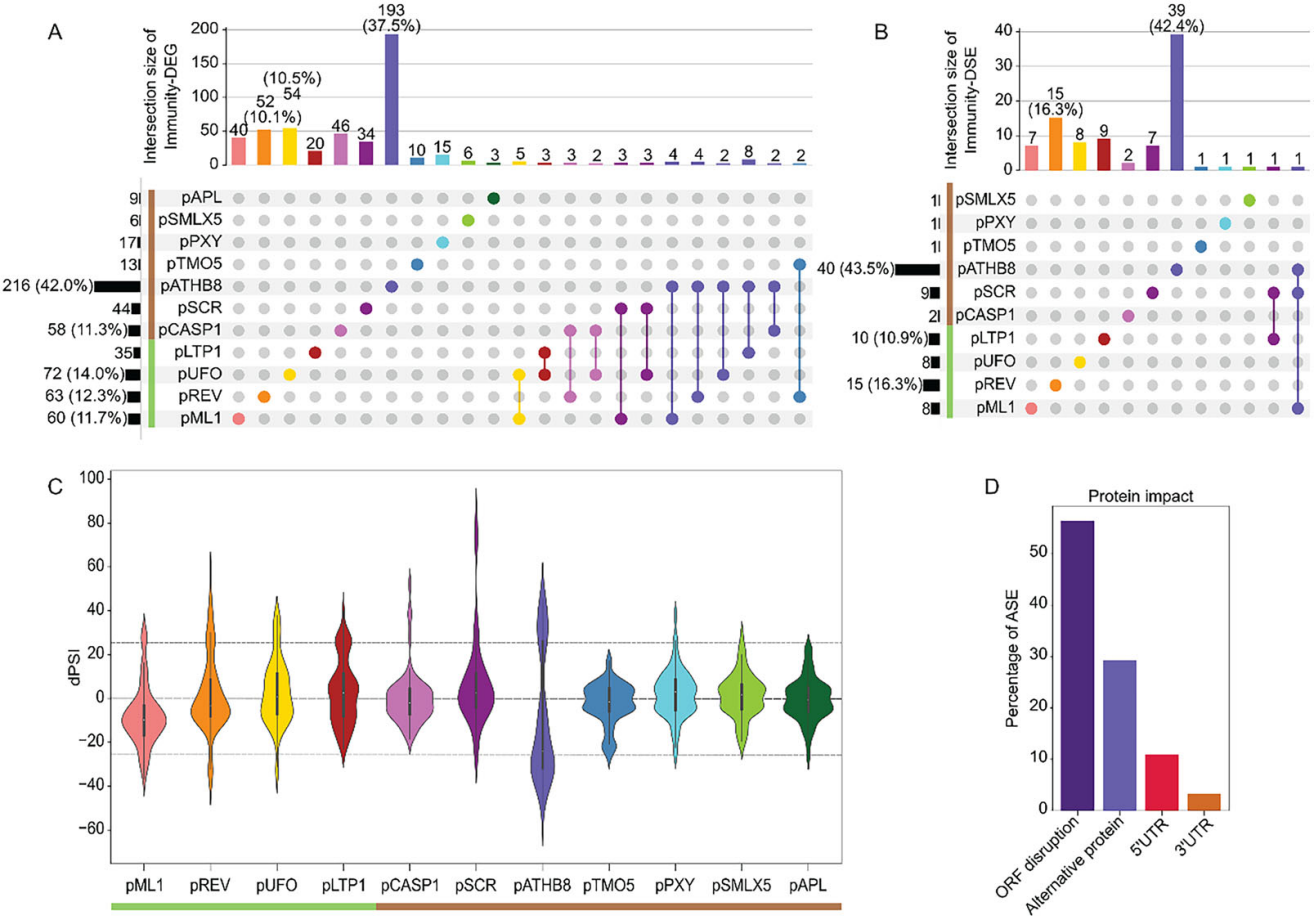
Differential splicing of immunity‐related genes is cell type‐specific. Upsetplots of immunity‐related (A) differentially expressed cell type‐specific genes (Immunity‐DEGs) and (B) differentially spliced cell type‐specific events (Immunity‐DSEs) across cell types. Green and brown bars represent aerial and root cell types, respectively. Violin plot of ∆PSI for the 92 Immunity‐DSEs in each cell type (C). ∆PSI represents an ASE's difference of PSI between a target tissue and the average of all the other tissues together (∆PSI = PSI_target_ − PSI_average_). Dotted lines show the thresholds used to determine if an ASE is significantly differentially spliced (25 < ∆PSI < −25). Predicted impact of the immunity‐DSEs on proteins (D). Bars represent the percentage of DSEs, which are predicted to cause either open reading frame (ORF) disruption, generate an alternative isoform, or overlap untranslated regions (UTRs).

The functional annotation analysis (Figure 2D) highlighted a strong tendency toward various responses to biotic and abiotic stresses among the DEGs. We sought to investigate the impact of cell identity on AS regulation of immunity‐related genes. We first compiled a list of plant immunity‐related genes (Table S2). From this list, we then identified 530 DEGs and 92 DSEs associated with genes involved in plant immunity, which we named immunity‐DEGs and immunity‐DSEs (Table S5). The 92 immunity‐DSEs originate from 71 immunity‐related genes (immunity‐DSGs). Unsurprisingly, p*ATHB8* shows once again the highest amount of DEGs and DSEs, with 193 immunity‐DEGs (Figure 5A) and 39 immunity‐DSEs (Figure 5B), while p*APL* shows only three immunity‐DEGs (Figure 5A) and no immunity‐DSE (Figure 5B). Besides p*ATHB8*, most root‐associated promoters present very few immunity‐DSEs. It is the case for p*TMO5*, p*PXY*, p*CASP1*, and p*SMLX5*, which have only one or two immunity‐DSEs (Figure 5B).

When looking at the ΔPSI (∆PSI = PSI_target_ − PSI_average_) of the immunity‐DSEs across cell types (Figure 5C), most of the immunity‐DSEs in this cell type are either over‐ or underrepresented, contrasting with the 10 other cell types where the ΔPSI median is closer to 0. Additionally, the functional analysis of the root cell types indicates that this promoter is mostly responsible for the immunity‐related annotations (Figure S5) found in the DEGs.

To assess whether the 92 immunity‐DSEs identified in our analysis have an impact on protein functionality, we used PastDB. This database generated by Martín et al. (2021) regroups AS profiles of Arabidopsis and associated features, like the predicted protein impact (Irimia et al., 2014). Using this database, we were able to extract the predicted protein impact of the immunity‐DSEs. Up to 56.5% of the immunity‐DSEs are predicted to cause ORF disruption (Figure 5D).

These observations align with the fact that, in addition to increased protein diversity, AS also plays a major role in gene translation regulation. In this instance, 60.4% of the immunity‐DSEs causing ORF disruption are upregulated, which can lead to the translation of a nonfunctional protein or to isoforms being processed through NMD, both resulting in a diminished quantity of functional protein. On the other hand, 39.6% of DSEs causing ORF disruption are downregulated, which implies a need for the other isoforms from the same genes to be upregulated. It is also important to note that at least 29.3% of immunity‐DSEs are predicted to generate alternative proteins (Figure S6).

**FIGURE 6:**
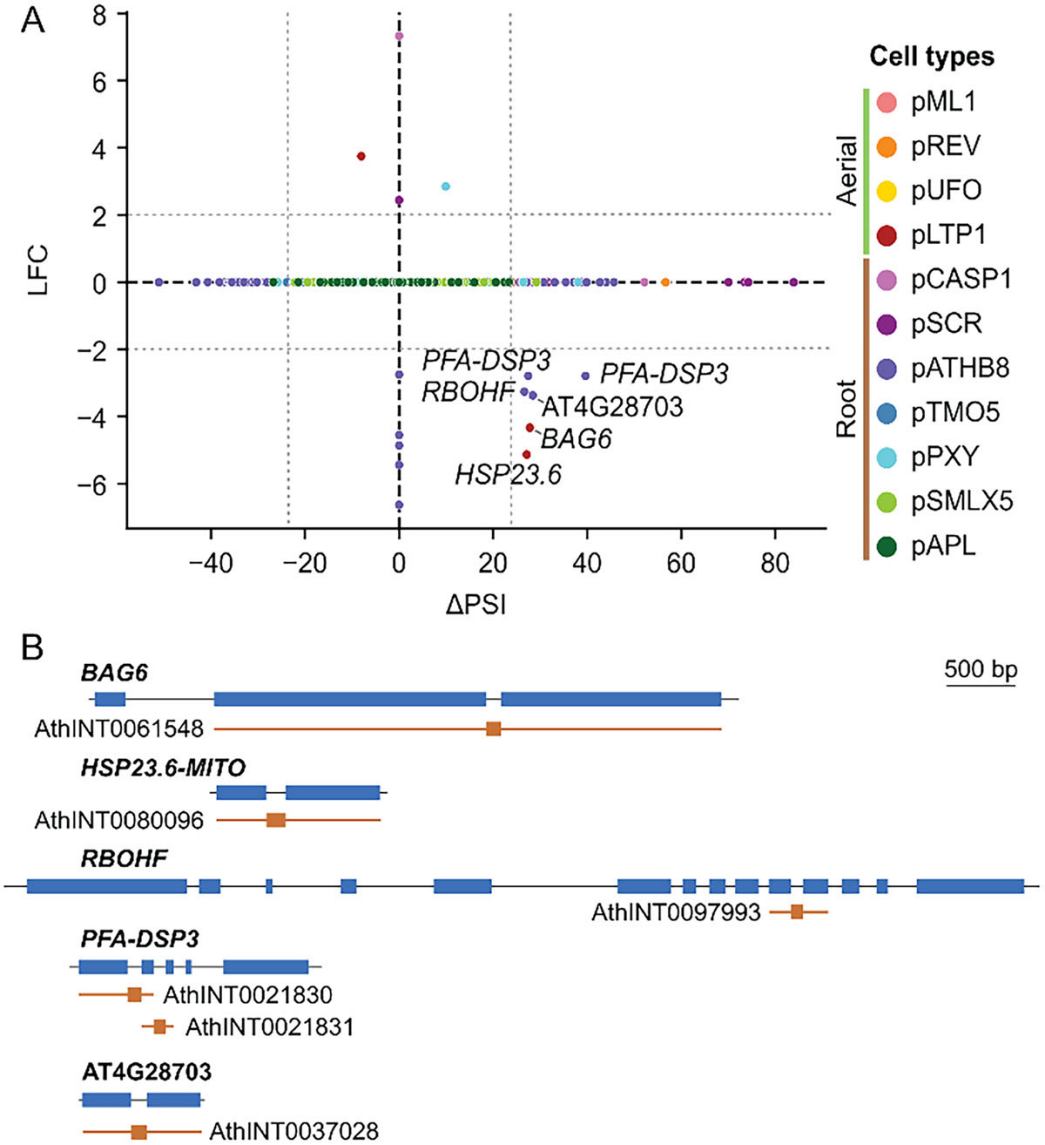
Gene expression and AS variation of immunity‐related genes. Comparison of gene expression and differential alternative splicing for the 92 immunity‐related DSEs in the 11 cell types (A). Gene expression is represented as fold change in a logarithmic scale (log 2 FC) (see Section 2 for details), while AS variation is represented as described previously with ΔPSI. Vertical dotted lines indicate the thresholds used to determine if an ASE is significantly differentially spliced (25 < ∆PSI < −25). Horizontal dotted lines indicate the thresholds used to determine if a gene is significantly differentially expressed (2 < log 2 FC < −2). DSEs that are differentially spliced and differentially expressed are identified by their gene name. Genomic representation of immunity DEGs that are also differentially spliced (B). Blue and black tracks represent exons and introns, respectively. Orange tracks show introns retained in the ASEs, orange lines indicate adjoining exons.

Among the genes from this group, the gene‐level expression varied significantly for five of them: *BCL‐2 associated athanogene 6* (*BAG6*), *plant and fungi atypical dual‐specificity phosphatase 3* (*PFA‐DSP3*), *mitochondrion‐localized small heat shock protein 23.6* (*HSP23.6‐MITO*), *A. thaliana* *respiratory burst oxidase homolog F* (*RBOHF*), and AT4G28703 (Figure 6). These genes showed repressed gene expression and upregulated ASEs. This is the case for *BAG6* (LFC = −4.33) and *HSP23.6‐MITO* (LFC = −5.13), for which one ASE, AthINT0061548 and AthINT0080096 respectively, present a significant upregulation (ΔPSI_AthINT0061548_ = 27.8 and ΔPSI_AthINT0080096_ = 27.13) in p*LTP1* (Figure 6, Figure S7, Table S6). *RBOHF*, *PFA‐DSP3*, and AT4G28703 also have negative gene expression and positive ΔPSIs, but this time in p*ATHB8* (Figure 6). Interestingly, AthINT0061548 from *BAG6*, AthINT0080096 from *HSP23.6‐MITO*, and AthINT0037028 from AT4G28703 are all predicted to result in alternative proteins (Table S6). This observation can also be made for *PFA‐DSP3*, whose gene‐level expression is reduced (LFC = −2.79) but displays two ASEs, AthINT0021830 and AthINT0021831, with a positive ∆PSI (∆PSI_AthINT0021830_ = 27.46, ∆PSI_AthINT0021831_ = 39.65) in p*ATHB8* (Figure 6). *PFA‐DSP3*'s AthINT0021831 is also predicted to result in an alternative protein (Table S6). Even upon reduction of the transcription level of specific genes, AS can lead to a significant change in isoforms, sometimes even leading to alternative proteins. Whether or not those alternative proteins are functional is yet to be determined. On the other hand, *RBOHF*'s AthINT0097993 and *PFA‐DSP3*'s AthINT0021830 are predicted to cause ORF disruptions (Table S5), which implies that, just like the inhibition of their gene expression, these ASE's upregulation would result in a decreased level of their corresponding functional protein. We also looked at those events and five other randomly selected Immunity‐DSEs in a long‐read sequencing isoform database (AtRTD3) (Zhang et al., 2022) to assess whether they had already been identified (Figure S8). Out of those 10 events, seven were found in AtRTD3. This observation suggests that our results are not biased by the method and bioinformatic tools used here.

Besides the five immunity‐related genes mentioned previously, 58 of the immunity‐related ASEs that are differentially spliced do not vary at the gene‐level expression in at least one cell type. Out of those 58, 12 are predicted to encode for an alternative protein. This implies that, even though transcription regulation is not altered for those genes, a shift in isoform population still occurs (Table S7).

Interestingly, our results accentuate the strong influence of AS on immunity‐associated genes, even leading to possible production of alternative proteins. Lastly, we also uncovered hints pointing toward the importance of cell identity on immunity regulation.

## 4 DISCUSSION

The process of AS in plants has proven to be an important tool in the adaptation to external and internal cues. While its mechanistic intricacies have been extensively analyzed, its broad impact on cell identity and plant immunity remains to be explored. In this study, we gathered data from 11 plant cell types originating from different organs, all collected through the same experimental process, to obtain high‐resolution AS data. Through our analysis, we highlighted the significance of AS on transcription regulation as well as on AS regulation itself, all in a cell type‐specific manner. Moreover, we showed that AS holds a role in the shaping of cell identity, especially in the columella cells. We also uncovered the impact of AS on immunity‐related genes in the columella cells. This study attempts to bridge the gap between cell identity, AS, and response to biotic stress. In addition, the dataset generated provides the scientific community with reliable tools to study cell identity and AS.

### 4.1 Alternative splicing regulation is independent from transcription

The first observation our findings indicate is that the genes targeted by expression regulation and the ones targeted by AS do not overlap (Figure 2A). Although AS occurs co‐transcriptionally, our results suggest that AS regulation is largely independent from gene expression regulation. This aligns with previous studies in which cell identity and stress triggered transcription and AS changes on nonoverlapping genes (Cheng et al., 2022; Li et al., 2013; Martín et al., 2021). This phenomenon does not seem to be generalized to plant development overall, as the Arabidopsis germline shares a considerable number of DEGs and alternatively spliced genes (Misra et al., 2023). The functional analysis of the DEGs highlighted tissue development, among others, as upregulated and immunity‐related processes as downregulated. On the other hand, genes targeted by AS are enriched for GO terms like DNA metabolic process, hence regulating gene expression. In addition, ASEs are also involved in RNA metabolic processes. This is expected as many splicing factors are themselves subjected to splicing, bringing another layer of regulation to AS (Jia et al., 2023; Lazar & Goodman, 2000).

### 4.2 Alternative splicing shapes cell identity

Through the use of specific promoters expressed on neighboring and sometimes even overlapping cell types, we managed to take a more refined look at the control AS holds over cell identity. Our dataset identified a greater number of cell type‐specific ASEs than previously done using the same analysis tools (Martin et al., 2021). This could be due to the process by which our dataset was generated, as all cell types were harvested similarly using the same induction system, whereas other studies have aggregated publicly available data. Although lower than the number of DEGs, we were still able to identify several cell type‐specific differentially spliced ASEs. Martin et al. (2021) also documented tissue and cell identity as impacting gene expression at a greater scale than AS. Our functional analysis also suggests that tissue development is particularly supported by gene expression (Figure 2D). While the influence of gene‐level regulation on cell identity is considerable, the expression profile of ASEs clustering according to cell types still hints toward the effect of AS on cell identity to be impactful (Figure 2B).

One of our main goals was to find nonoverlapping regulated ASEs. Even though some root cell types studied here are either very close to each other in terms of identity or represented by multiple promoters, our analysis still yielded a few specific DSEs. We suspect that the difference in AS regulation between those cell types is due to the maturation stage of the root cells. The phloem cells, for example, are represented by both p*APL* and p*SMLX5*. The main difference between the two promoters resides in the developmental phase of the phloem cells they represent. The same rationale applies to p*SCR* and p*CASP1*, both expressed in the endodermis. Our analysis was able to capture the subtle changes in AS regulation necessary between different developmental stages of corresponding cell types. Hence, it identified genes better suited for future cell type‐specific studies, as very few cell type‐specific promoters available are truly cell type‐specific. Just like the promoters used here, many will have overlapping expression across neighboring cell types. Therefore, the present study provides a list of new genes and promoters to develop new cell type‐specific biological tools (Table S3).

The shoot and root apical meristem provided substantial differences in pathways (Figure S5) and intensity of gene‐level expression and AS regulation. This is to be expected as even though both perform active mitotic cell division, the two meristems are involved in largely different tissue growth (Xue et al., 2020). While most aerial cell types differentially express a somewhat similar number of genes, the shoot apical meristem (SAM) central zone (*pREV*) is definitely subjected to a more intense ASE regulation (Figure 3B). This is corroborated by Tian et al. (2019), who noted the same effect when comparing the different zones of the SAM. AS is known to be involved in maintaining the SAM zone boundaries and cell division pattern through proteins like SKIP (Li et al., 2022). Nevertheless, the broader implication and importance of AS in the SAM, more particularly in the central zone, have yet to be investigated. Similarly, the quiescent center of the root apical meristem also shows a certain degree of AS regulation. We hypothesize that AS may hold a role in maintaining the stem niches and therefore in the developmental plasticity of meristems.

### 4.3 Alternative splicing, cell identity, and immunity‐associated genes are interconnected

To fill part of the gap between plant immune response, cell identity, and AS, we examined immunity‐related genes among cell type‐specific DEGs and DSGs. The distinction between transcription and AS regulation is also true for immunity‐related genes. At first glance, the majority of the immunity‐related processes seem transcriptionally regulated, as shown by the functional analysis of the DEGs (Figure 2D). However, when separating root and aerial regulated ASEs, we noticed that aerial ASEs are enriched for plant hypersensitive response (Figure S5). The role of AS in immunity was proven multiple times through the study of key factors of plant defense (Golisz et al., 2021; Howard et al., 2013; Liu et al., 2022; Xie et al., 2023), and yet the global extent of its impact on resistance has yet to be discussed. Almost up to 10% of the cell type‐specific regulated ASEs are immunity‐related even in the absence of an actual defense stimulus. This implies that AS could participate in maintaining the growth to immune response balance. According to the predicted protein impact, half of the defense ASEs are expected to cause ORF disruption (Figure 5E). The aforementioned observation indicates that AS might act as another layer of immune transcriptional regulation. It possibly negatively regulates the accumulation of immunity‐related proteins in a context in which they are not needed while still maintaining a basal gene expression level to quickly produce the complete protein when needed. Still, the 30% of immunity‐related ASEs resulting in actual alternative protein isoforms also imply that AS potentially has a direct impact on immune signaling pathways in a cell type‐specific manner. As we know, the initiation of a defense response is very consuming to the plant (Smakowska et al., 2016). Hence, the switch of energy allocated to growth versus defense needs intense regulation. Our result suggests that AS plays an important role at different levels to guard the induction of plant defense.

### 4.4 Columella cells defense system is regulated through alternative splicing

One of the main highlights of this analysis comes from p*ATHB8* cells, which present the highest number of DEGs and DSGs (Figure 3A,B). These results could be partially attributed to the way the differentially spliced and differentially expressed analyses are inherently built. These analyses exclusively uncover nonoverlapping genes between conditions, and since many of the promoters used are expressed in overlapping cell types (Figure 1), we expected only a small number of DSGs or DEGs. Among the root cell types, the promoters used here, p*ATHB8* and p*SCR*, are the only ones specific to the columella and the quiescent center, respectively. Hence, p*ATHB8* yielded more specific genes and isoforms than the other promoters did. Moreover, we safely assume that most of those regulated genes and isoforms are specific to the columella cells. Following this logic, we would also expect p*SCR* to exhibit a higher number of specific regulated genes and isoforms. While not as much as p*ATHB8*, p*SCR* still presents more specific genes and isoforms than other root promoters do. The quiescent center being less represented than the endodermis in terms of quantity is probably one of the reasons p*SCR* does not show a higher number of DSEs and DEGs. However, this hypothesis only partially explains why p*ATHB8* and the columella in particular seem to undergo intense gene and isoform regulation. Additionally, it does not clarify why nearly all of these genes are mostly downregulated.

Columella cells play multiple physiological roles, including but not limited to the perception of gravity (Staehelin et al., 2000), production of mucilage (Maeda et al., 2019), acting as a physical barrier, and involvement in plant defense (Driouich et al., 2013; Hawes et al., 2000). Those cells also undergo tight coordination of stem cell production and cell release at the outer root cap layer. This process is crucial for proper root development. The columella is thus composed of three underlying cell types: the columella stem cells, the columella differentiated cells, and the border‐like cells, which ultimately go through programmed cell death (Kumar & Iyer‐Pascuzzi, 2020). Being responsible for such major functions could justify the need for a more intense regulation of gene expression and isoform splicing. We hypothesize that the imminent cell death could lead to major repression of gene expression. Additionally, columella cells and the root cap in general also act as barriers protecting the apical meristem (Driouich et al., 2013; Fortier et al., 2023; Hawes et al., 2000; Hawes et al., 2016). The mucilage they produce is, among other things, composed of antimicrobial proteins and reactive oxygen species (Driouich et al., 2021; Maeda et al., 2019). The border‐like cells in particular are responsible for the formation of the root extracellular trap, a biofilm‐like structure acting as the first physical line of defense of root meristems (Driouich et al., 2021; Hawes et al., 2000). According to our findings, the columella cells immune mechanism is subjected to intense regulation through gene expression, but also AS. The functional analysis of the DEG in p*ATHB8* (Figure S5) also indicates that the columella is responsible for the immunity‐related annotations. We postulate that the crucial role of the border‐like cells in plant defense is strongly controlled by AS and is responsible for the distribution of the immunity‐related isoforms observed in p*ATHB8*. While more extensive work is necessary to explore further on the subject, our result certainly highlights the unsuspected importance of AS in the columella cells.

AS is a conserved process that enables eukaryotes to reach a higher level of transcriptome and proteome complexity. This mechanism is essential in the adaptation to various stresses and developmental stages of plants. To better understand the significance of AS on those numerous stimuli, many focused on the study of specific factors of plant development and stress tolerance. We, however, sought to observe AS's broader impact on plant growth and plant immunity. Here, we managed to establish links between AS, cell identity, and immunity‐related genes. Our results also highlighted the unsuspected importance of AS in the columella cells. AS might play an important part in the balance between the growth and defense states. Although the global Arabidopsis AS landscape upon elicitation of a defense response still needs to be investigated, our analysis gives an interesting insight into the interconnection between AS, immunity‐related genes, and cell identity.

## AUTHOR CONTRIBUTIONS

**Teura Barff**: Conceptualization; data curation; formal analysis; investigation; methodology; writing—original draft; writing—review and editing. **Ingrid Berenice Sanchez Carrillo**: Investigation; methodology. **Valeria Paola Parra Gutiérrez**: Methodology. **Mélodie B. Plourde**: Conceptualization; methodology; project administration; resources; writing—review and editing. **David L. Joly**: Conceptualization; supervision; writing—review and editing. **Hugo Germain**: Conceptualization; formal analysis; funding acquisition; investigation; methodology; project administration; resources; writing—review and editing.

## CONFLICT OF INTEREST STATEMENT

The authors declare no conflicts of interest.

## Supporting information



Table S1: Samples and replicates sequencing information

Table S2. List of defense‐related genes used to identify the immunity‐DEGs and DSEs

Table S3. Cell type‐specific genes and ASE

Table S4. Expression of housekeeping genes in p*ATHB8*

Table S5. Cell type‐specific immunity related genes and ASE

Table S6. Immunity‐related splicing variant of interest

Table S7. Immunity‐related DSE that do not vary at the gene expression level

Supplementary Figures: Fig. S1 to S8

## Data Availability

Raw sequencing data were submitted to Genbank under Bioproject accession number PRJNA1097566.
