# Supplementary material for: Comprehensive mapping of Arabidopsis alternative splicing landscape reveals key insights into plant development and immunity
Source: Plant Genome. 2025 Mar 28;18(2):e70022. doi: 10.1002/tpg2.70022 (PMC11953613; doi:10.1002/tpg2.70022)
Supplement: Supplementary file 1 [file TPG2-18-e70022-s004.pdf]

Comprehensive mapping of Arabidopsis alternative splicing landscape reveals key insights into plant development and immunity.

Supplementary material.

Table S1: Samples and replicates sequencing information
Table S2. List of defense-related genes used to identify the immunity-DEGs and DSEs
Table S3. Cell type-specific genes and ASE
Table S4. Expression of housekeeping genes in p*ATHB8*
Table S5. Cell type-specific immunity related genes and ASE
Table S6. Immunity-related splicing variant of interest
Table S7. Immunity-related DSE that do not vary at the gene expression level

Supplementary Figures: Fig. S1 to S8

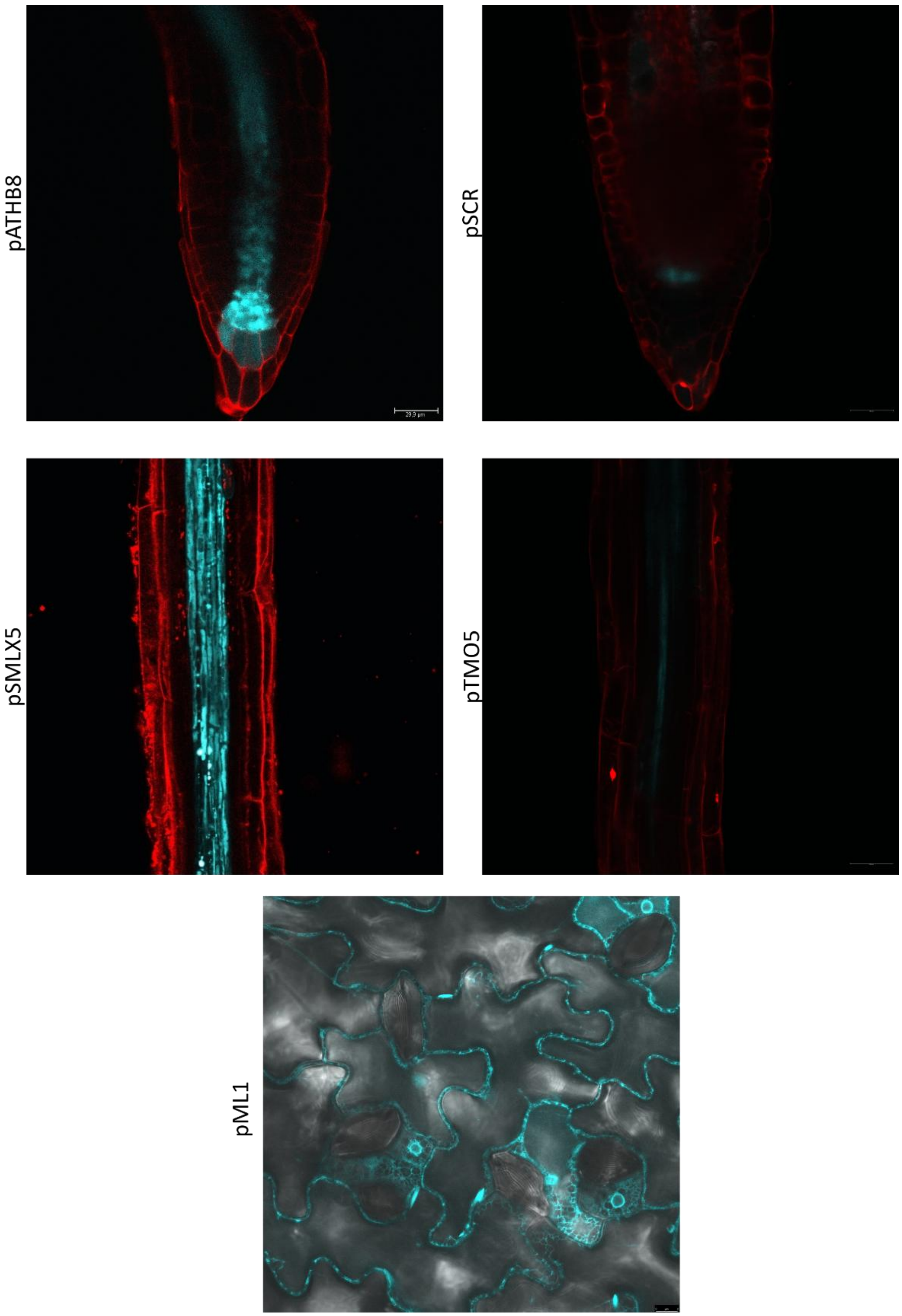

Supplementary Figure S1. Confocal images of root and leaf cell type.

Confocal imaging of roots (pATHB8, pSCR, pSMLX5 and pTMO5) and leaves (pML1) from transgenic plants dyed with PI (red) observed with Excitation/Emission wavelengths of 488 nm/590-660. mTurquoise expression (blue) is acquired with Excitation/Emission wavelengths of 405 nm/460-520 nm. Scale bar: 29.9 µm

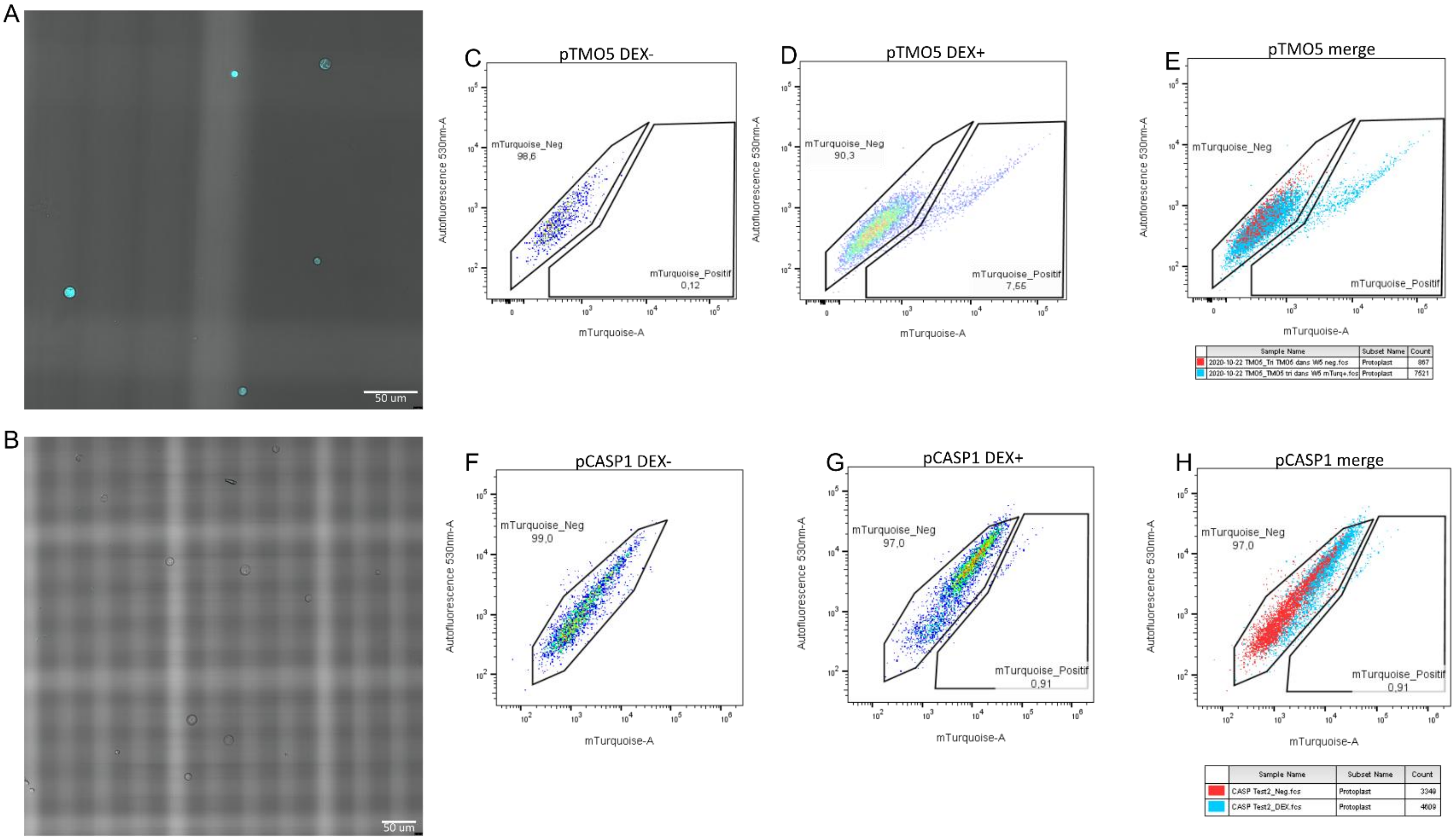

Supplementary Figure S2. Protoplast cell sorting.

Confocal imaging of protoplasts after cell sorting. Protoplasts originating from p*SMLX5* transgenic plants collected as expressing mTurquoise2 (A) and protoplasts from the same sorting experiment collected as negative for mTurquoise2 expression (B). Scale bar: 50 µm. FACS plotting of events obtained with a laser of 405 nm and a bandpass filter of 528/545 nm (mTurquoise-A) against events obtained with a laser of 488 nm and a bandpass of 527/532 nm (Autofluorescence-530nm-A) (C-H). Dot plot of protoplasts extracted from p*TMO5* plants grown on non-DEX medium, used to gate the mTurquoise2 negative events (C). Dot plot of protoplasts from p*TMO5* plants grown on DEX medium (D). Merge of the non-DEX grown plants (red dots) and DEX grown plants of p*TMO5* (blue dots) (E). Same plots are shown for p*CASP1* transgenic plants (F-H).

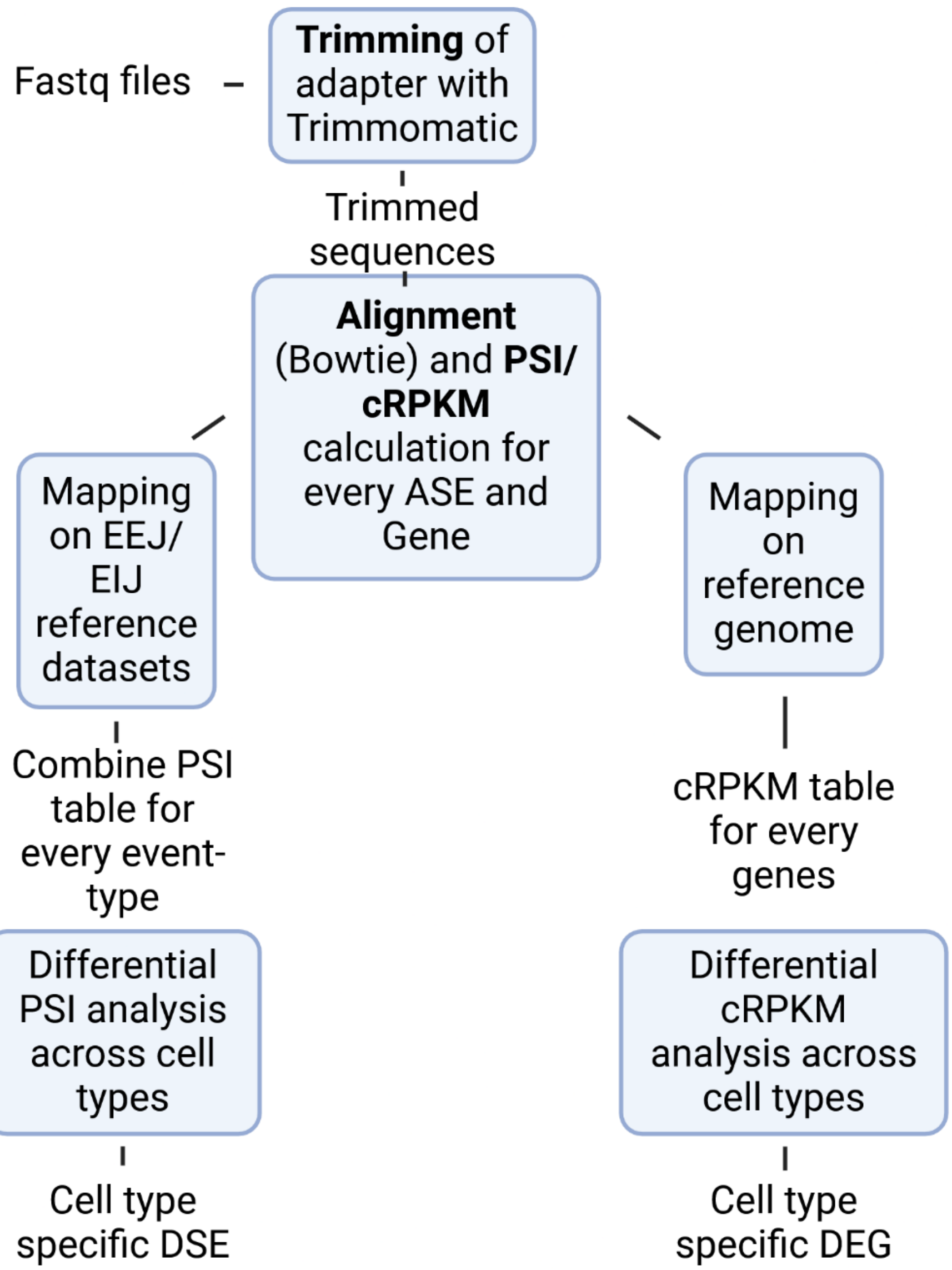

Supplementary Figure S3. Vast-tool isoform and gene analysis workflow.
EEJ: Exon-Exon Junction, EIJ: Exon-Intron Junction

Supplementary Figure S2. Isoform and gene analysis workflow

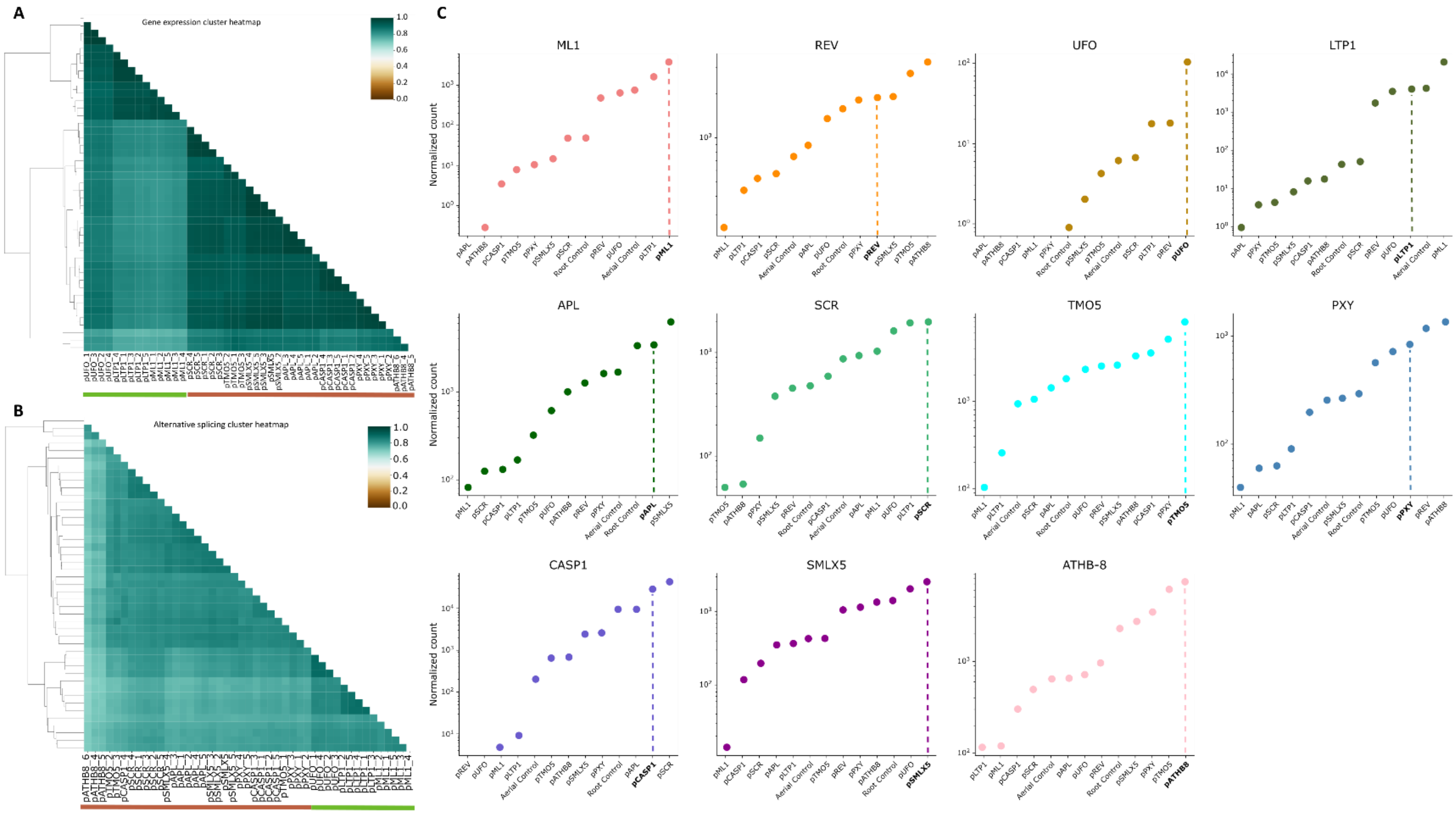

Supplementary Figure S4. Protoplast processing has minimal effect on their identity.
Spearman cluster heatmap of cRPKM (A) and PSI values (B) across replicates. Clustering method used is 'average'. Normalized read counts of the genes controlled by the cell type-specific promoters used in this study (C). Each panel corresponds to a specific gene. X-axis shows the transgenic plants expressing mTurquoise2 under different promoters. X-axis order is rearranged to show the promoter with the lowest to the highest count from left to right. Dotted line is used to highlight the corresponding promoter of the gene observed.

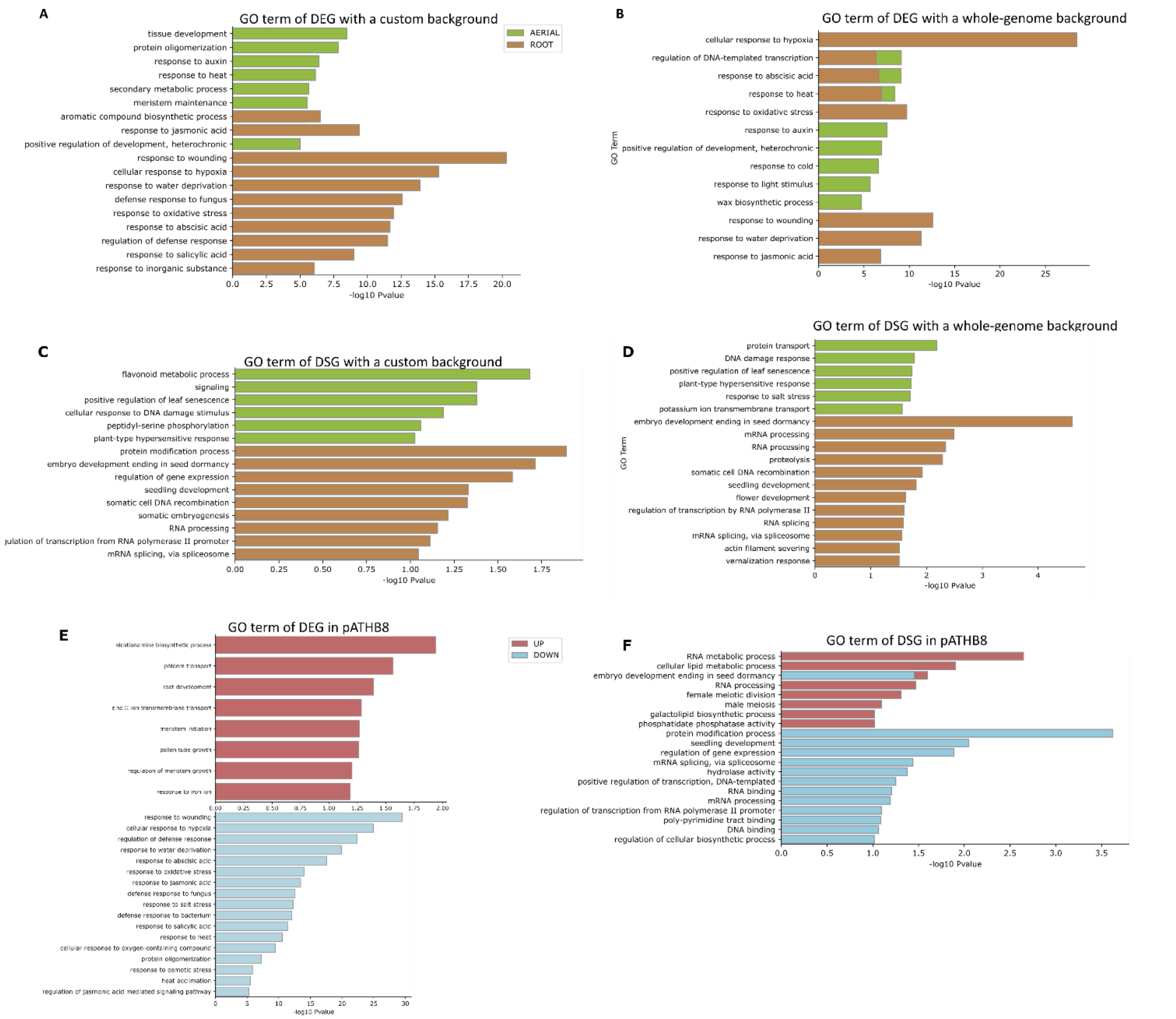

Supplementary Figure S5. Supplementary functional analysis.

Enriched GO term analysis of DEGs obtained with a custom background (A) and with a whole-genome background (B) and of DSGs obtained with a custom background (C) and with a whole-genome background (D) in which aerial (green) and root (brown) promoters were separated. Enriched GO terms of DEGs (E) and DSGs (F) from p*ATHB8*. down (blue) and up (red) regulated GO terms are represented. Bars indicate –log10 of the DAVID's EASE score modified *P*-value. The comparison between GO terms obtained with a whole-genome or a custom background shows little difference. The analysis done with the whole-genome background still presents mostly immunity-related GO term.

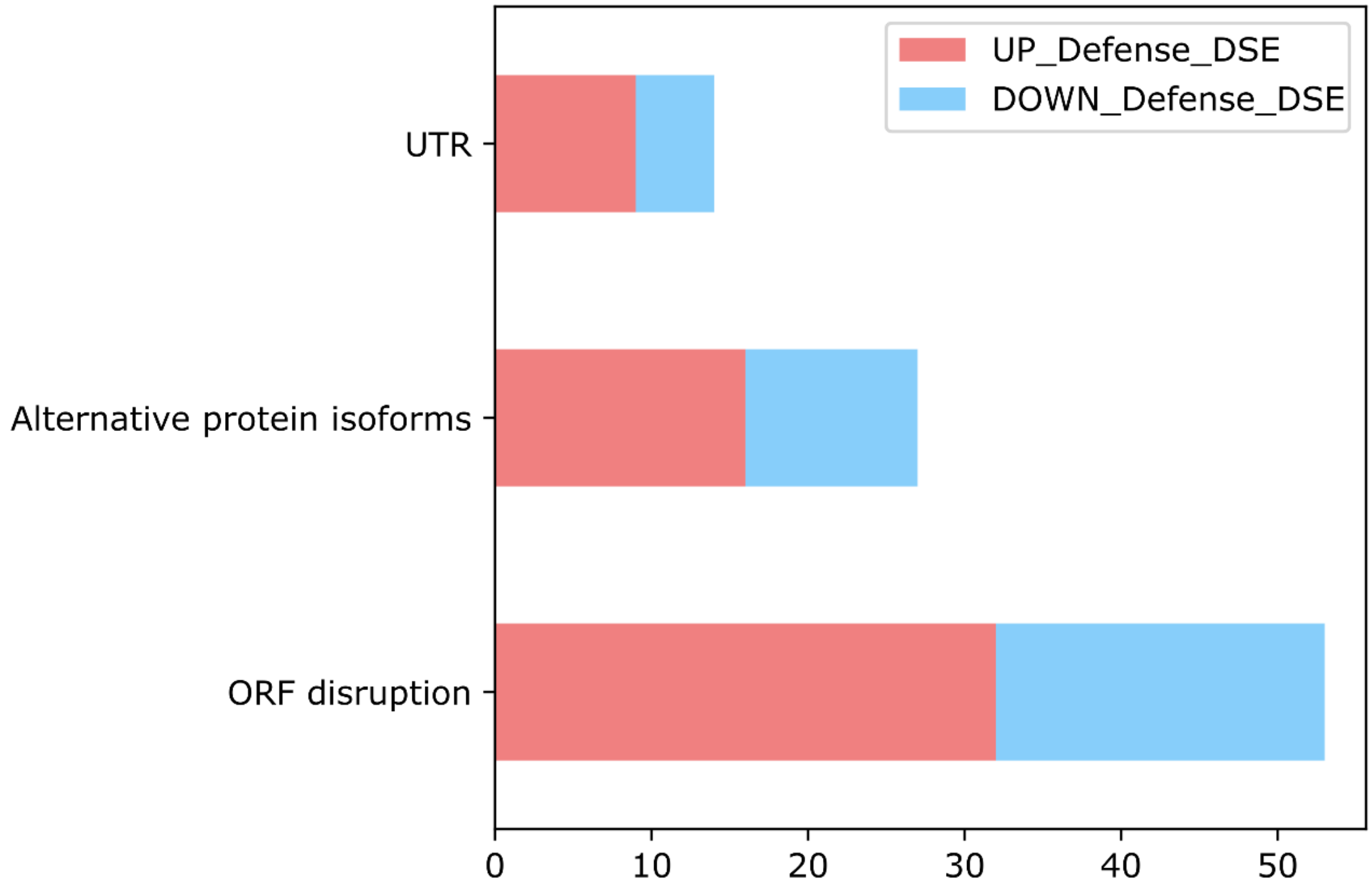

Supplementary Figure S6. Predicted protein impact of the immunity-related DSE separated in up- or down-regulation. Predicted impact of the immunity-DSEs on proteins. Bars represent the percentage of DSEs, which are predicted to cause either ORF disruption, generate an alternative isoform, or overlap UTRs. Red and blue represent up and downregulated DSE, respectively.

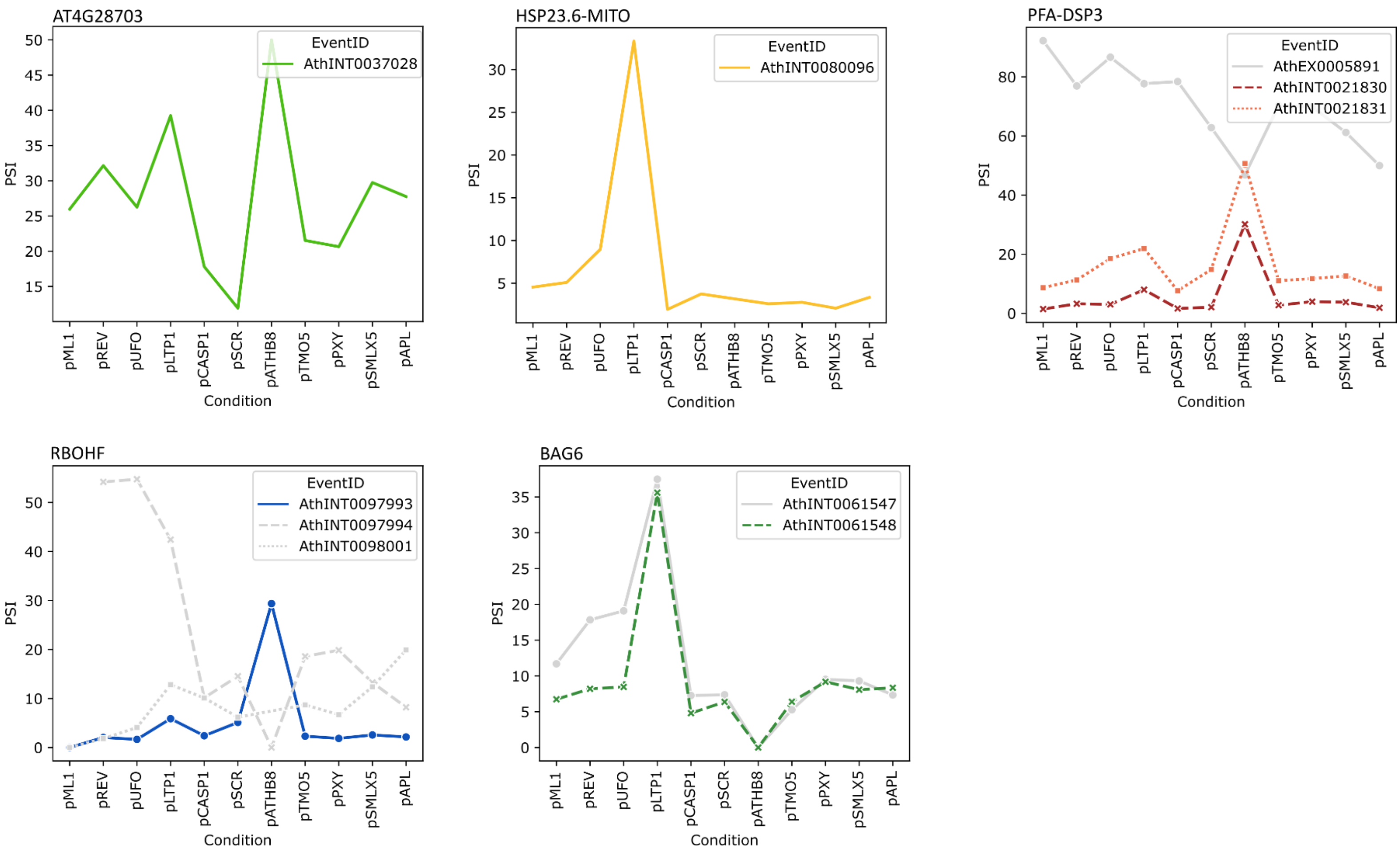

Supplementary Figure S7. All splice variants from the immunity-related genes that are differentially spliced and differentially expressed. PSI variation across cell types of all the splicing variants from the immunity-related genes identified in Fig.6. Colored lines show the splice variants that are identified as significantly alternatively spliced. The variants that show a big variation of PSI but are colored in grey did not pass the quality filters described in methods to be identified as alternatively spliced.

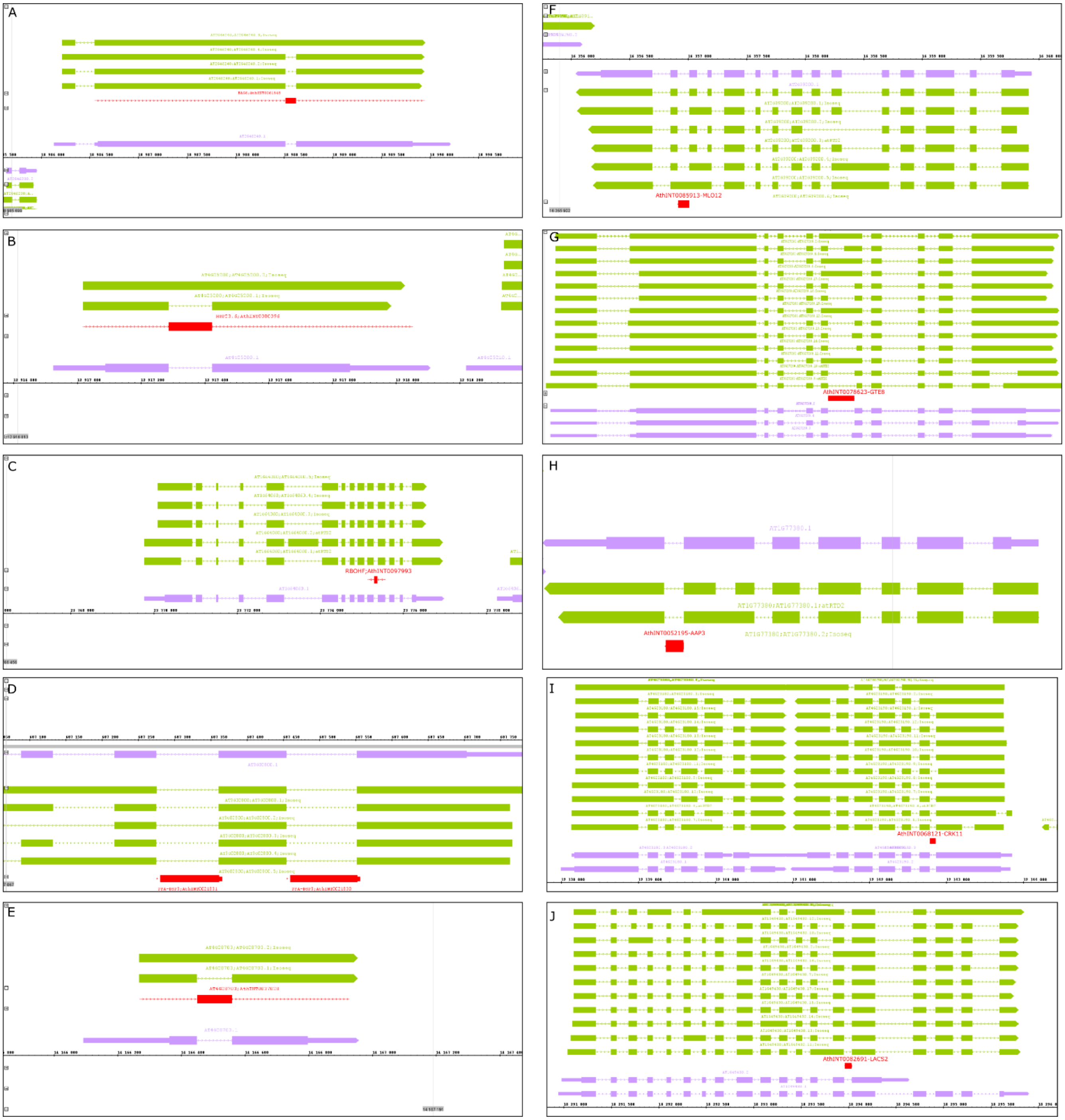

Supplementary Figure S8. Comparative analysis of events of interest from our study with isoforms from AtRTD3. Genomic tracks of the 6 Immunity-DSEs that are also differently expressed (A-E). Genomic tracks of 5 randomly selected Immunity-DSEs (F-J). Green tracks are isoforms data from the Iso-seq database, AtRTD3. Red tracks represent the events found in our study, with the coordinates from PASTDB. Purple tracks are genomic data from Araport. Note that tracks from PASTDB correspond only to AS events, not whole isoform. Additionally, only the AS event of interest is displayed, meaning that PASTDB contains more unshown AS events for each gene present in this figure.
